# Mesoporous Silica Nanoparticles With Customized Drug Ratio/Loading for Effective Treatment of Gemcitabine-Resistant Pancreatic Tumors

**DOI:** 10.1002/anbr.202500255

**Published:** 2026-03-23

**Authors:** Tamanna Binte Huq, Sudip Kumar Dam, Doaha Awad, Punnya Anil Kumar Jeeja, Farzana Ferdous, Juan L. Vivero-Escoto

**Affiliations:** 1 Department of Chemistry, University of North Carolina at Charlotte, Charlotte, North Carolina, USA; 2 Chemistry and Nanoscale Science Program, University of North Carolina at Charlotte, Charlotte, North Carolina, USA; 3 Department of Biological Sciences, University of North Carolina at Charlotte, Charlotte, North Carolina, USA; 4 Center for Biomedical Engineering and Science, University of North Carolina at Charlotte, Charlotte, North Carolina, USA

**Keywords:** combination therapy, controlled release, gemcitabine resistance, mesoporous silica nanoparticles, pancreatic cancer

## Abstract

Pancreatic ductal adenocarcinoma (PDAC) is one of the most lethal cancers, with a 5-year survival rate below 13% and limited treatment options due to rapid metastasis and pronounced chemoresistance. Gemcitabine (Gem) remains a first-line chemotherapy agent; however, its clinical efficacy is hindered by poor cellular uptake, incomplete activation, and acquired drug resistance. To address these limitations, we develop redox-responsive mesoporous silica nanoparticles (MSNs) for the mono- and codelivery of Gem and Cisplatin (cisPt). In this work, we tune the loading and ratio of Gem and cisPt within MSNs. To evaluate the therapeutic potential of MSN-based delivery system in Gem-resistant (GR) PDAC cell lines, we establish murine (GR-KCM) and human (GR-BxPC3) cell models and further evaluate the efficacy in highly GR human cell lines (AsPC1 and HPAFII). In vitro studies demonstrate that Gem-MSNs (10%wt) and Gem-cisPt-MSN (10:9%wt) exhibit the strongest cytotoxicity, even in GR models. Notably, the combination Gem-cisPt-MSN (18:9%wt) induces pronounced S-phase cell cycle arrest, apoptosis, and reactive oxygen species. These findings underscore the potential of MSN-based drug delivery systems to enhance chemotherapy efficacy in treatment-refractory PDAC.

## 1 | Introduction

Pancreatic ductal adenocarcinoma (PDAC) remains one of the most lethal malignancies, with limited therapeutic success largely due to the aggressive nature of the disease, early metastasis facilitated by vascular and neural invasion, late-stage diagnosis due to the absence of early diagnostic biomarkers, and widespread resistance to conventional chemotherapy regimens [1–4]. Although newer combination therapies such as FOLFIRINOX (folinic acid, 5-fluorouracil, irinotecan, and oxaliplatin) and gemcitabine (Gem) plus nab-paclitaxel have improved clinical outcomes for select patients [5, 6], the effective delivery of multiple drugs at the specific place and in a timely manner remains a significant challenge. Nanoparticle-based carriers, such as liposomes, polymeric, inorganic, and metallic nanoparticles have been explored to address these limitations [7]. However, many of these carriers suffer from limitations including instability, off-target and premature drug release, and physicochemical incompatibilities [8–11]. In the context of combination therapy, additional challenges arise from the need to coload drugs with different chemical properties at defined ratio, achieving synchronized or sequential release profiles, and encapsulate sufficiently high doses of multiple drugs are major limitations of traditional drug delivery systems [12, 13]. To tackle these issues liposomes and polymeric nanoparticles have been used; however, precise coloading of hydrophobic/hydrophilic drugs and high loading are still remain challenges [14–16].

Mesoporous silica nanoparticles (MSNs) have emerged as promising platforms for targeted drug delivery in oncology due to their high surface area, tunable pore sizes, biocompatibility, and functionalization flexibility. MSNs enable coloading of multiple therapeutic agents in ratiometric proportions, allowing for simultaneous or sequential drug release [9–11, 17–19]. Prior reports have described Gem + Paclitaxel (PTX) in lipid-coated MSN platform; however, these systems did not demonstrate the ability to tune Gem/PTX ratios or to enhance cytotoxicity through resistantbypass mechanism in PDAC cells [15, 20]. Our group developed a Gem-cisPt-MSN platform for the efficient delivery of Gem and cisplatin (cisPt) for the treatment of PDAC [21, 22]. The system relies on the selective chemical attachment of prodrugs derived from Gem and cisPt that are responsive to the highly reducing environment inside the PDAC cells and/or tumor microenvironment. We demonstrated the loading of Gem/cisPt with a timely controlled release of the parent drugs. The Gem-cisPt-MSN system was successfully tested in vitro and in vivo PDAC models [22]. In the present work, we further expand this platform by demonstrating its capacity for ratiometric engineering to achieve variable Gem/ cisPt weight loadings, high precision and reproducibility in loading efficiency and enhanced therapeutic performance against Gem resistant PDAC cell lines.

The emergence of Gem resistance remains a major therapeutic challenge [7, 8]. This resistance stems from both phenotypic plasticity, such as epithelial-mesenchymal transition (EMT), and various intracellular enzymatic and molecular adaptations. While EMT is a dynamic process critical for embryogenesis and tissue repair, in PDAC it is co-opted to drive tumor progression and drug resistance [4, 23]. During EMT, cell–cell adhesion is weakened, allowing cancer cells to invade the basement membrane, enter the bloodstream, and eventually establish metastases through mesenchymal-epithelial transition. This shift is accompanied by downregulation of epithelial markers such as E-cadherin, certain cytokeratins, occludin, and claudin and upregulation of mesenchymal markers such as Vimentin, N-cadherin, and fibronectin, as well as transcription factors including Snail, Slug, Twist, and Zeb1, which repress epithelial characteristics and promote stemness, motility, and resistance to apoptosis [24–26].

Gem, an S-phase-specific deoxycytidine analog, depends on transmembrane transporters such as human equilibrative nucleoside transporter (hENT)1–2 and human concentrative nuceloside transporter (hCNT)1–3 for cellular uptake [27]. Once internalized, Gem requires phosphorylation by deoxycytidine kinase (dCK), nucleoside monophosphate kinase, and nucleoside diphosphate kinase to become active and incorporate into DNA, making the expression of these kinases critical for its efficacy [27, 28]. In contrast, elevated levels of ribonucleotide reductase (RR) sustain deoxynucleotide pools, reducing Gem incorporation and limiting its ability to inhibit replication and cell cycle progression. To overcome drug resistance associated with Gem, recent efforts have focused on codelivery strategies that improve therapeutic synergy and increase cytotoxicity [27]. Notably, combining Gem with platinum-based agents such as cisPt has been shown to reduce resistance, suppress tumor growth, and promote apoptosis [21]. cisPt, a DNA-damaging agent, causes replication fork stalling and double-strand breaks, while Gem inhibits RR and terminates DNA chains. Together, they synergistically induce S-phase toxicity, disrupt cell cycle progression, and increase apoptosis [8, 28].

**FIGURE 1 |:**
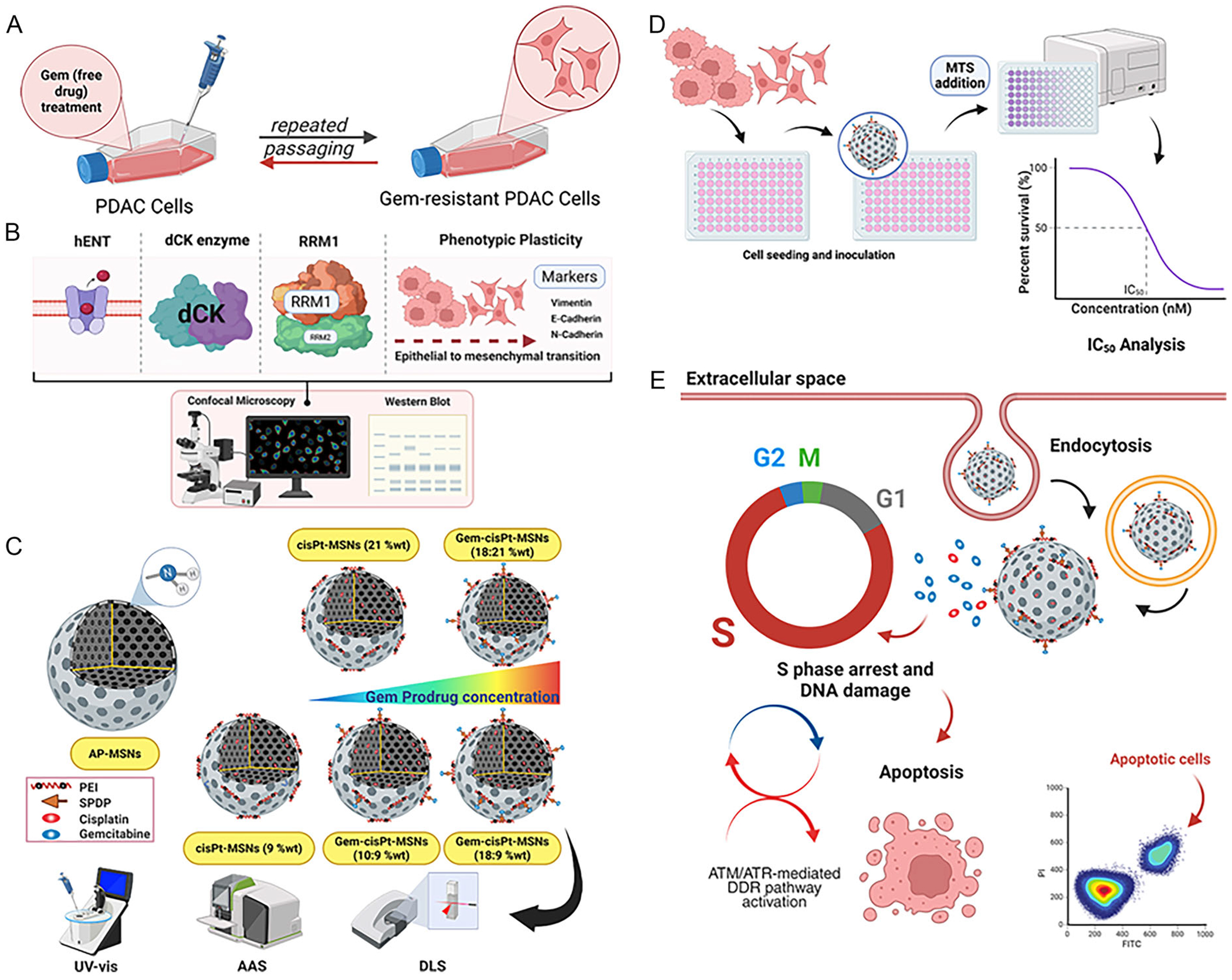
Schematic illustration of the work carried out in this paper. (A) Development of GR PDAC cell lines using the stepwise drug selection method. (B) Characterization of the phenotypic plasticity properties and various intracellular enzymatic and molecular adaptations associated with Gem resistance. (C) Tuning of the loading ratio of Gem and cisPt to MSNs and characterization of the nanoparticles. (D) Evaluation of the cytotoxic effect of Gem-cisPt-MSN platform using the MTS assay. (E) Testing the cell death mechanisms by apoptosis and cell-cycle assays associated with the Gem-cisPt-MSNs.

In this study, we have developed murine (KCM) and human (BxPC3) Gem-resistant (GR) PDAC cell lines using a stepwise drug selection approach (Figure 1A) [29–32]. The phenotypic plasticity of these cells was evaluated by investigating the levels of three EMT biomarkers, Vimentin, E- and N-cadherin through Western blot and confocal microscopy. Moreover, key proteins involved in Gem uptake and metabolism including hENT1, dCK, and ribonucleotide reductase M1 subunit (RRM1) were investigated by Western blot and RT-qPCR (Figure 1B). The observed upregulation of EMT-associated markers and altered expression of Gem-related proteins confirm the development of GR PDAC cell lines. We also tuned the loading of Gem and cisPt in MSNs at varied weight percentages (Figure 1C). The physicochemical properties of the nanoparticles were characterized using dynamic light scattering (DLS) and Z-potential. The amount of chemically available amines was quantified by ninhydrin assay, and the loading of Gem and cisPt were determined using AAS and UV–vis spectrometry. The findings demonstrated a reliable fabrication method with consistent repeatability. We used the GR-KCM and GR-BxPC3 to evaluate the therapeutic outcome of the different ratios of Gem-cisPt-MSNs (Figure 1D,E). The cytotoxic impact of these materials was determined using the MTS, which showed that Gem MSN (10%) and Gem-cisPt-MSNs (10:9%wt) exhibited the strongest cytotoxicity. Apoptotic and cell cycle arrest effects were determined using the FITC-Annexin V/ Propidium Iodide (PI) and cell-cycle assays, respectively. These assays demonstrated that Gem-cisPt-MSNs (18:9%wt) exhibited the highest apoptotic effect and enhanced cell-cycle arrest even in resistant models.

## 2 | Results and Discussion

### 2.1 | Development of Gemcitabine-Resistant KCM and BxPC3 Models for PDAC

**FIGURE 2 |:**
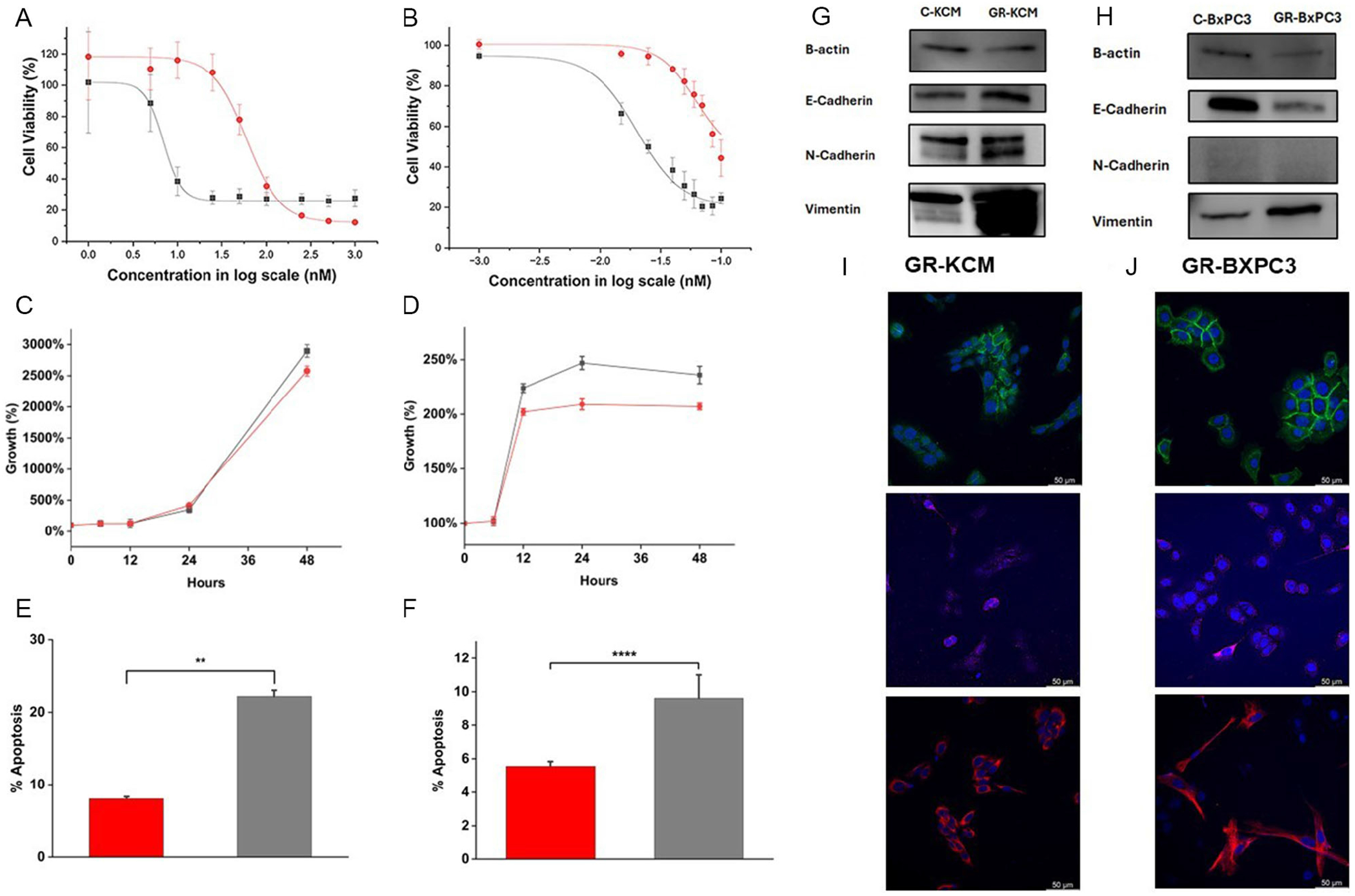
Drug-response plots for (A) GR-KCM (red) and C-KCM (gray) cells and (B) GR-BxPC3 (red) and C-BxPC3 (gray) cells. Comparison of growth between (C) GR-KCM (red) and C-KCM (gray) cells and (D) GR-BxPC3 (red) and C-BxPC3 (gray) cells in the absence of Gem. Apoptosis of (E) GR-KCM (red) and C-KCM (gray) cells and (F) GR-BxPC3 (red) and C-BxPC3 (gray) cells upon Gem treatment. (G and H) Representative Western blots for E-cadherin, N-cadherin, and Vimentin in C/GR-KCM and C/GR-BxPC3 cell lines, respectively. (I and J) EMT marker expression in GR-KCM and GR-BxPC3 cells, respectively: cells were stained with DAPI (blue), E-cadherin (green/top), N-cadherin (magenta/middle), and Vimentin (red/bottom). Statistical comparisons were conducted using one-way ANOVA followed by Tukey’s multiple comparison test. Significance levels are denoted as follows: ****p ≤ 0.0001, ***p ≤ 0.001, **p ≤ 0.01, and *p ≤ 0.05.

A stepwise drug selection method was used to develop the murine (GR-KCM) and human (GR-BxPC3) GR PDAC cell lines [29–32]. The PDAC cell lines were cultured in the presence of Gem for about 4–5 months, allowing for the gradual development of resistance to the drug. To account for any morphological or physiological changes attributable to prolonged passaging, control cultures of KCM (C-KCM) and BxPC3 (C-BxPC3) cells were maintained under identical conditions, but in the absence of Gem exposure. To verify the development of resistance to Gem in these cell lines, cell viability (MTS) assay was conducted to obtain the IC_50_ values based on the drug-response curves (Figure 2A,B). The IC_50_ values for GR-KCM and C-KCM were 61.5 ± 6.4 nM and 6.9 ± 6.4 nM, respectively (Table S1). In the case of GR-BxPC3 and C-BxPC3, the IC_50_ values obtained were 166.9 ± 26.0 nM and 56.5 ± 7.7 nM, respectively. The IC_50_ values indicated that GR-KCM and GR-BxPC3 need 9 and 3 times more Gem drug as compared with the control cell lines. In addition, in the absence of Gem, the proliferation of GR-KCM and GR-BxPC3 cells was slower than C-KCM and C-BxPC3 cells (Figure 2C,D). Finally, the apoptosis induced by Gem under similar conditions was also lower in GR-KCM and GR-BxPC3 cells than in C-KCM and C-BxPC3 cells (Figure 2E,F). Slower growth and lower apoptosis values than the corresponding control cell lines are features that have been previously reported for GR cell lines [31]. These results clearly indicate the development of resistance to Gem treatment in both KCM and BxPC3 cell lines.

To examine the changes on the phenotypic plasticity properties of GR-BxPC3 and GR-KCM cells, EMT markers were analyzed through Western blotting and confocal microscopy. For GR-KCM cells, Western blotting shows an increase in Vimentin and both E- and N-cadherin as compared with C-KCM (Figures 2G and S1). In the case of GR-BxPC3 cells, Western blotting indicates an increase in Vimentin, and a decreasing trend in both N- and E-cadherin as compared to C-BxPC3 (Figures 2H and S1). The expression of these proteins demonstrates phenotypic plasticity towards EMT in GR-KCM and GR-BxPC3 cells. Upregulation of Vimentin and N-cadherin promotes stemness, motility, and resistance to apoptosis [33–35]. Reduction in E-cadherin weakens cell–cell adhesion, which is a key step in promoting metastasis through EMT. However, recent literature shows that E-cadherin in promoting tumor progression and invasiveness, which may explain the observed results in KCM cells [36]. Confocal micrographs of GR-KCM and GR-BxPC3 cells stained with antibodies against these markers (Vimentin, E- and N-cadherin) revealed localization patterns consistent with EMT (Figure 2I,J). N-cadherin staining (magenta) was predominantly localized around the cell body and at cell–cell junctions. Vimentin staining (red) showed strong cytoplasmic expression, indicative of mesenchymal phenotype. E-cadherin expression (green) was redistributed around the cell membrane, suggesting a loss of epithelial characteristics. In a similar way, confocal micrographs of C-KCM and C-BxPC3 cells were stained with these markers (Figures S1). Overall, these results support the hypothesis that GR-KCM and GR-BxPC3 cells have undergone EMT, as evidenced by their marker expression profiles.

**FIGURE 3 |:**
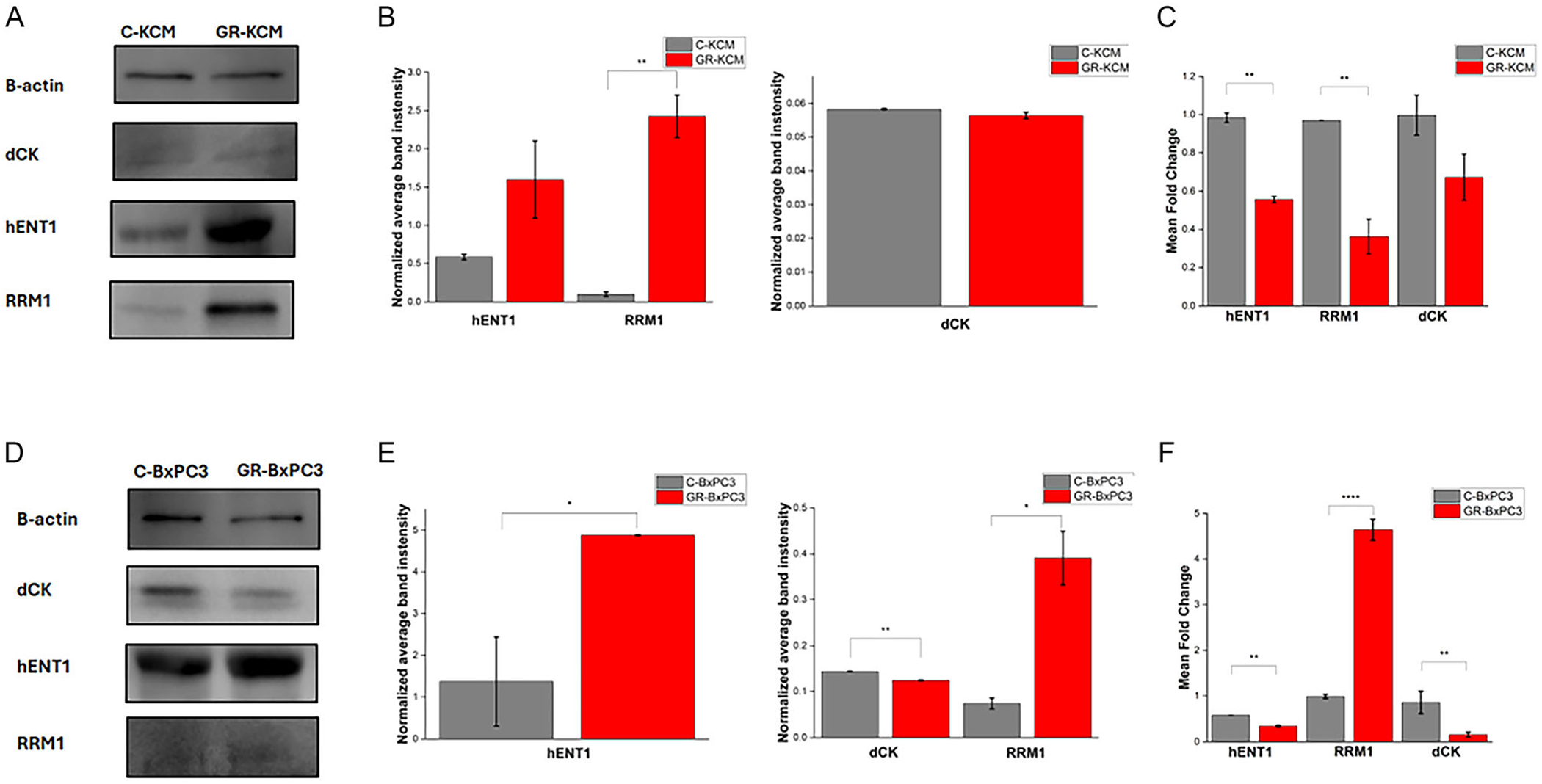
Representative Western blots showing protein levels of dCK, hENT1, and RRM1 in C/GR-KCM (A) and C/GR-BxPC3 (D) cell lines. Quantification of protein levels of dCK, hENT1, and RRM1 in C/GR-KCM (B) and C/GR-BxPC3 (E) cell lines and corresponding RT-qPCR results in (C) C/GR-KCM (F) C/GR-BxPC3 cells. Data presented as mean ± standard deviation (SD), *n* = 2 for Western blot data and *n* = 3 for RT-qPCR data. Statistical comparisons were conducted using one-way ANOVA followed by Tukey’s multiple comparison test. Significance levels are denoted as follows: ****p ≤ 0.0001, ***p ≤ 0.001, **p ≤ 0.01, and *p ≤ 0.05.

Studies on the cell mechanisms of Gem resistance have identified some resistance-related genes including hENT1 (transporter), dCK (phosphorylation enzyme), and RRM1 (contributes to an increase in the endogenous deoxynucleotides) [37–39]. Using Western blotting, we evaluated the expression of these proteins in GR-BxPC3 and GR-KCM cells (Figure 3). An upregulation of hENT1, the primary nucleoside transporter for Gem, was observed in both GR-KCM and GR-BxPC3 cells as compared with the control cells (Figure 3A,B,D,E). Studies looking at the hENT1 levels in human PDAC cells including BxPC3 also observed an increase in hENT1 in Gem treated cells [40]. Although in other PDAC cells, hENT1 changes may lead to a higher resistance to Gem, the levels of hENT1 can vary due to nucleoside deprivation and increased proliferation between cell lines [41, 42]. Interestingly, RT-qPCR for GR-KCM and GR-BxPC3 shows downregulation of hENT1 (Figure 3C,F). The difference seen in protein and mRNA levels may be due to several factors such as complex post-transcriptional regulation, varying protein lifespans, and measurement variability of both molecules [43, 44]. dCK is the first key kinase in the Gem mechanism and is considered the rate-limiting step for the active diphosphorylated and triphosphorylated forms of Gem [27, 37]. For both GR-KCM and GR-BxPC3 cells, Western blot and RT-qPCR data demonstrate a downregulation of dCK (Figure 3A–F). The decrease in dCK levels in both GR-KCM and GR-BxPC3 suggests less formation of the active diphosphorylated and triphosphorylated forms of Gem, and therein less incorporation of the drug into the DNA to exert its cytotoxic effects [45]. Another key player in the Gem resistance mechanism is the RRM1 protein. RRM1 contributes to an increase in the endogenous deoxynucleotides, which compete with Gem [46]. The expression of RRM1 in both GR-KCM and GR-BxPC3 cells is upregulated (Figure 3A,B,D,E). According to the RT-qPCR, RRM1 is downregulated in GR-KCM and upregulated in GR-BxPC3 (Figure 3C,F). Our results indicate that GR-KCM and GR-BxPC3 exhibit similar changes in expression of hENT1, dCK, and RRM1 proteins to those previously reported in the literature, which point out to typical mechanisms developed by GR cell lines.

Overall, our findings demonstrated that the developed GR-KCM and GR-BxPC3 cell lines exhibit increased tolerance to Gem. Several well-known mechanisms of Gem resistance, including decreased expression in dCK and increased expression in RRM1, were observed in these cell lines. Additionally, both GR-KCM and GR-BxPC3 cell lines have undergone EMT. Collectively, these results confirm the successful development of murine GR-KCM and human GR-BxPC3 cell lines.

### 2.2 | Optimizing Drug Loading and Release Behavior in MSN-Based Nanocarriers

**FIGURE 4 |:**
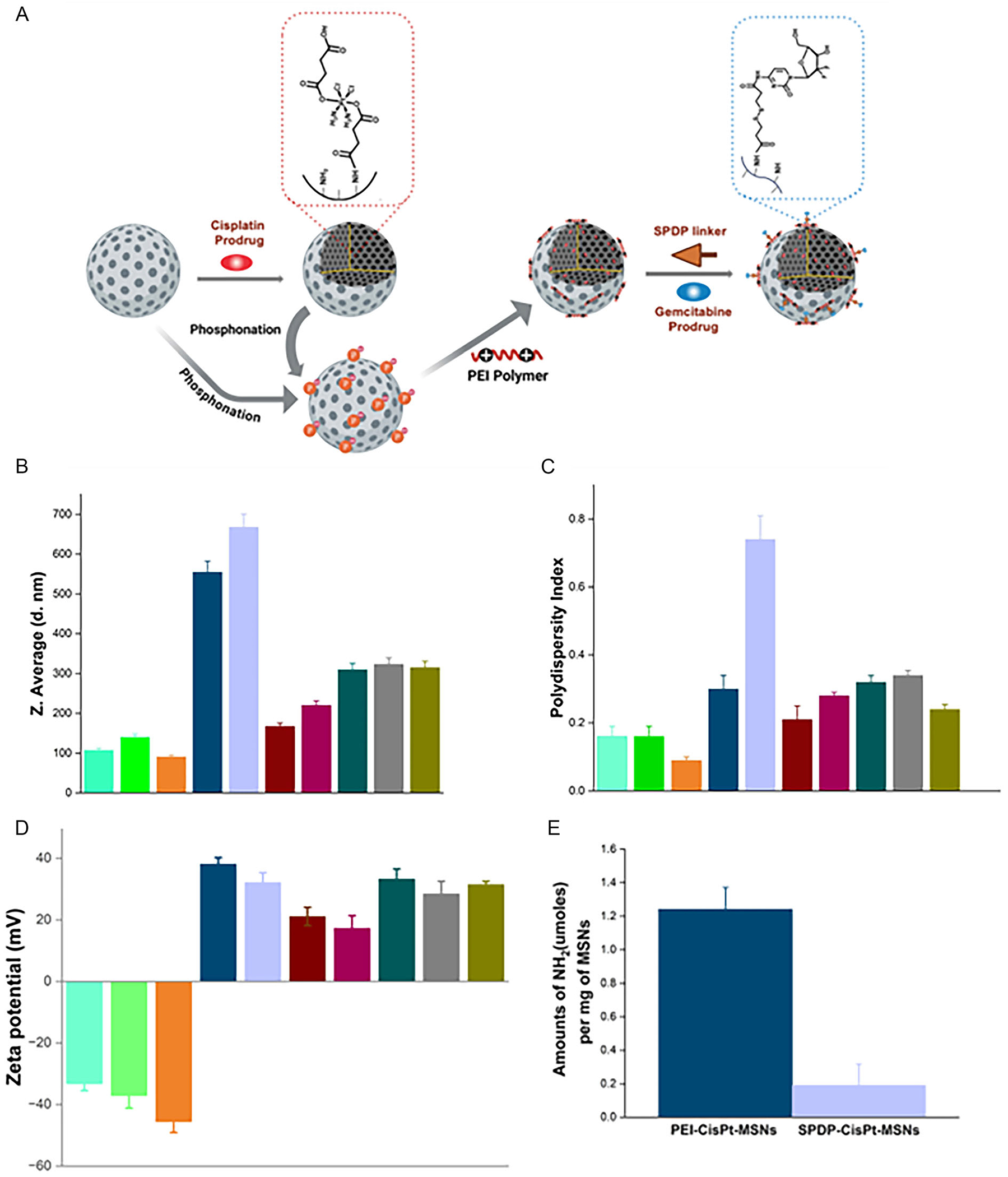
(A) Schematic representation of the overall synthesis of Gem-cisPt-MSNs. (B) DLS analysis showing the Z-average *H_D_*. (C) PDI. (D) ζ-potential measurements of all MSN formulations. Error bars represent SD (*n* = 8 for all the functionalized MSNs; *n* = 4 for drug-conjugated combinations). (E) Quantitative determination of surface available amines. Color code: AP-MSNs (Cyan); cisPt-MSN (lime green); Phos-cisPt-MSN (burnt orange); PEI-cisPt-MSN (navy blue); SPDP-cisPt-MSN (lavender); Gem-MSN (10%wt) (brick red); Gem-MSN (18%wt) (burgundy); Gem-cisPt-MSN (10:9%wt) (dark green); Gem-cisPt-MSN (18:9%wt) (gray); and Gem-cisPt-MSN (18:21%wt) (olive).

We have shown the advantageous properties of MSNs for codelivering Gem and cisPt in controlled ratio and spatiotemporal fashion [44, 47]. Nevertheless, our previous reports only showed the use of MSNs for a fixed ratio of Gem:cisPt. Herein, by tuning the initial concentration of cisPt or Gem prodrugs, we controlled the adsorption equilibrium loading factor of the cisPt and Gem prodrugs to control the amount of prodrug chemically loaded to MSNs. By using this approach, the following materials were fabricated: cisPt-MSNs (9 and 21%wt), Gem-MSNs (10 and 18%wt), and Gem-cisPt-MSNs (Gem:cisPt = 10:21, 10:9, and 18:9). The following general protocol was used for the synthesis of those materials (Figure 4A) [21]. First, the prodrugs of cisPt and Gem were synthesized using a two-step process adapted from reported methodologies with slight modifications from our lab. The details for the synthesis and characterization are depicted in the supporting information. MSNs were synthesized by a surfactant-templated approach with cocondensation of AP-TES. The cisPt prodrug was further loaded to MSN via a coupling reaction mediated by 1-ethyl-3-(3-dimethylaminopropyl) carbodiimide hydrochloride (EDC). This material was modified with phosphonates to render on the surface stronger negatively charged. cisPt-MSN was further coated with polyethyleneimine (PEI) (1.8 kDa) to afford PEI-cisPt-MSNs. After PEI coating, the nanoparticles underwent modification with N-succinimidyl 3-(2-pyridyldithio) propionate (SPDP). We varied the degree of SPDP modification to fine-tune the amount of Gem prodrug attached to the surface of the material. Through a disulfide displacement reaction between SPDP and Gem prodrug, the final material was obtained, Gem-cisPt-MSNs. By tuning the cisPt prodrug concentration, the amount of SPDP conjugated to PEI-cisPt-MSNs, and the concentration of Gem prodrug chemically attached to the MSNs, we created a series of MSN formulations with precise cisPt:Gem ratios (Table S2). Experimental details for the conditions used to synthesize the MSN formulations are depicted in the supporting information. The amount of cisPt and Gem prodrugs loaded was determined by atomic absorption spectroscopy (AAS) and UV– vis spectroscopy, respectively. The conjugated cisPt prodrug was 9.2 ± 3.4 (*n* = 4) or 21.1 ± 1.2%wt (*n*=4) and the Gem prodrug was 10.9 ± 1.3 (*n*=4) or 18.7 ± 0.7%wt (*n*=4) (Table S2).

A physical analysis including DLS, ξ-potential, surface adsorption/ desorption (BET/BJH) properties and transmission electron microscopy (TEM) was performed. N2 isotherms were used to determine the surface area (BET method) and pore volume/ diameter features of the initial MSN material. The hydrodynamic diameter (*H*_D_) of AP-MSNs is 107.6 ± 15 nm with a narrow polydispersity index (PDI) of 0.16 ± 0.03 and a ξ-potential of −33.2 ± 2.3 mV, consistent with a well-dispersed and stable colloidal system in phosphate-buffered saline (PBS). The surface area was 799.0 ± 77.0 m^2^/g, pore volume of 1.45 ± 0.2 cc/g, and average pore diameter of 2.3 ± 0.1 nm (Figure S2 and Table S3). TEM image shows a spherical MSN with a diameter of 62.3 ± 5.1 nm (Figure S2). The step-by-step modification of the AP-MSNs was followed by determining the *H*_D_, PDI, and ξ-potential.

Chemical loading of cisPt prodrug resulted in minimal change in the *H*_D_ (140.3 ± 4 nm), ξ-potential (−37.1 ± 4.1 mV) and PDI (0.16 ± 0.03) due to attachment of the molecule to the internal surface of MSNs (Figure 4B–D and Table S4). Modification of the cisPt-MSN surface with phosphonate groups produced a change in the ξ-potential (−45.6 ± 3.6 mV), but without major changes in the colloidal stability of the nanoparticles *H*_D_ (91.4 ± 5 nm) and PDI (0.09 ± 0.01). Further functionalization with PEI afforded major changes in *H*_D_ (554.2 ± 4 nm), PDI (0.30 ± 0.04) and ξ-potential (+38.1 ± 2.1 mV). The presence of amine groups, which are protonated under physiological conditions, changed the surface charge of the nanoparticle; however, imperfect coating with PEI resulted in aggregation. The amine groups from PEI were quantified using a ninhydrin assay, 1.25 ± 0.13 μmol of amine per mg of MSNs were determined (Figure 4E). To load Gem prodrug to PEI-cisPt-MSNs, a NHS-based crosslinker reagent SPDP is chemically conjugated to the PEI’s free amines. The addition of SPDP resulted in major increase on particle aggregation, HD (667.3 ± 11 nm), PDI (0.74 ± 0.10) due to the presence of hydrophobic molecules on the surface of the nanoparticles. The amount of free amine groups was reduced to 0.19 ± 0.13 μmol of amine per mg of MSNs (86.4% reduction) as an indication of the successful functionalization with SPDP (Figure 4E). Following modification with SPDP, Gem prodrug was conjugated via a disulfide exchange reaction to afford the final material Gem-cisPt-MSNs.

The amount of Gem-loaded had an impact on the colloidal stability of the final formulation. Gem-MSN (10%wt) (HD = 167.0 ± 4.3 nm and PDI = 0.21 ± 0.04) showed better colloidal stability than Gem-MSN (18%wt) (*H*_D_ = 220.0 ± 7.0 nm and PDI = 0.28 ± 0.01). In the case of Gem-cisPt-MSN materials, the impact on the colloidal stability is even higher, Gem-cisPt-MSN (10:9%wt) (*H*_D_ = 310.0 ± 3.0 nm and PDI = 0.32 ± 0.02); Gem-cisPt-MSN (18:9%wt) (*H*_D_ = 323.0 ± 4.0 nm and PDI = 0.34 ± 0.01); Gem-cisPt-MSN (18:21%wt) (*H*_D_ = 315.0 ± 5.0 nm and PDI = 0.24 ± 0.01) (Figure 4B–D and Table S4).

We then assessed long-term colloidal stability by monitoring hydrodynamic size and PDI over 20 h in complete media (Figure S3). There was a clear difference in the aggregation between the monotherapy nanoparticles Gem-MSNs and the combined therapy Gem-cisPt-MSNs. The *H*_D_ for the Gem-MSNs (10%wt) and Gem-MSNs (18%wt) is 169 and 225 nm with PDI of 0.24 and 0.29 respectively. Both parameters remained constant during the whole 20 h. On the contrary, the *H*_D_ for all the Gem-cisPt-MSN formulations is between 310 and 340 nm with a DPI that varies from 0.24 to 0.32. These results clearly indicate that Gem-cisPt-MSN materials showed a higher aggregation than Gem-MSNs. The colloidal stability of nanoparticles can impact their interaction with cancer cells by influencing their cellular uptake [48, 49].

The rational design of our nanoplatform enabled the coconjugation of two physicochemically distinct drugs at different loading efficiencies and the fabrication process demonstrated high reproducibility across multiple independent batches (Table S2). The stimuli-responsive release of the MSN platform employing chemical conjugation of Gem/cisPt drugs via redox-responsive linkages has already been reported by our group [21]. It is expected that a similar performance for the systems developed in this work.

### 2.3 | Influence of Drug Ratios on Cytotoxic Efficacy Across PDAC Cell Lines

To evaluate the cytotoxic properties of the MSN formulation the drug–response curve was determined by the MTS assay and the IC_50_ values calculated using a sigmoidal model (Figure S4 and Tables S5–S8). For this evaluation, in addition to C-KCM, GR-KCM, C-BxPC3, and GR-BxPC3 cells, we included AsPC1 and HPAF II cell lines. Both AsPC1 and HPAF II have shown a high resistance to treatment with Gem [29]. We performed MTS assays comparing seven MSN formulations including the monotherapy (Gem-MSNs = 10 and 18%wt; cisPt-MSNs = 9 and 21%wt) and the combinations (Gem-cisPt-MSNs = 10:9, 18:9 and 18:21%wt).

**FIGURE 5 |:**
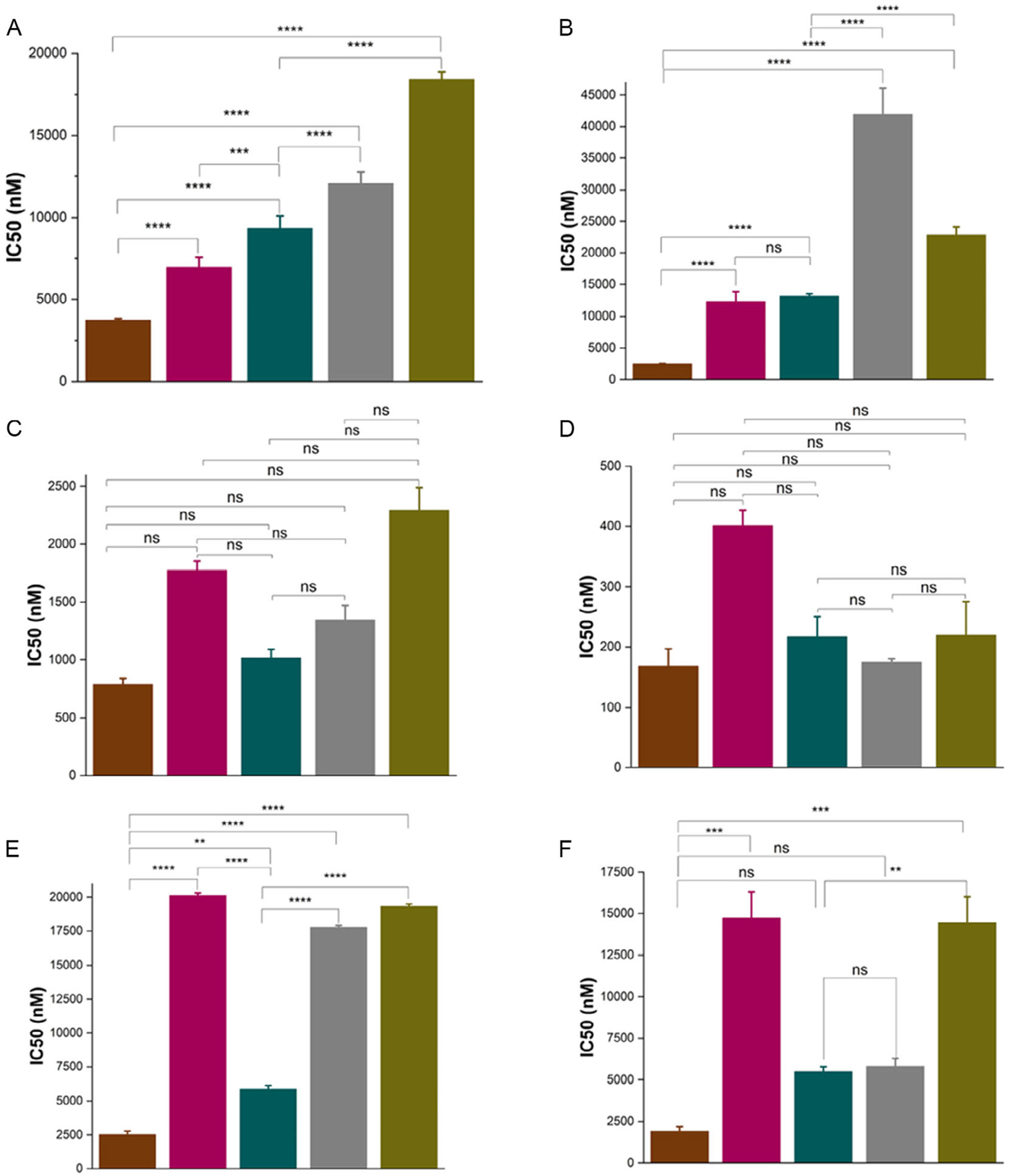
IC_50_ values for mono- and dual-drug loaded MSNs across pancreatic cancer cell lines. (A) GR-BxPC3, (B) C-BxPC3, (C) GR-KCM, (D) C-KCM, (E) AsPC1, and (F) HPAF-II. Color code: Gem-MSN (10%wt) (brick red); Gem-MSN (18%wt) (burgundy); Gem-cisPt-MSN (10:9%wt) (dark green); Gem-cisPt-MSN (18:9%wt) (gray); Gem-cisPt-MSN (18:21%wt) (olive). All data are presented as mean ± SD from three independent experiments (*n* = 3). Statistical comparisons were conducted using one-way ANOVA followed by Tukey’s multiple comparison test. Significance levels are denoted as follows: ****p ≤ 0.0001, ***p ≤ 0.001, **p ≤ 0.01, *p ≤ 0.05, and not significant (ns) for *p* > 0.05.

The Gem-MSN (10%wt) formulation achieved the lowest IC50 values for GR-KCM (787.3 ± 49.7 nM) and GR-BxPC3 (3749.3 ± 105.1 nM) (Figure 5A–C and Table S5). The best combination was Gem-cisPt-MSNs (10:9%wt) with IC50 values of 1016.7 ± 73.5 nM and 9363.1 ± 817.8 nM for GR-KCM and GR-BxPC3, respectively. There was no statistical difference for the cytotoxic effect between Gem-MSN (10%wt) and Gem-cisPt-MSNs (10:9%wt) for GR-KCM; however, in the case of GR-BxPC3, Gem-MSN (10%wt) performed better. In the case of C-KCM and C-BxPC3 a similar trend is observed, with Gem-MSN (10%wt) formulation achieving the lowest IC_50_ values for C-KCM (168.3 ± 28.5 nM) and C-BxPC3 (2505.0 ± 38.0 nM) (Figure 5B–D). Gem-cisPt-MSNs (10:9%wt) were the best combination to kill C-BxPC3 cells with a IC_50_ value of 13 141.0 ± 380.1 nM. Nevertheless, all the combinations showed no statistical differences to reduce the viability of C-KCM cells (Figure 5D and Table S5). Interestingly, the viability assay for C-BxPC3 cells resulted in higher IC50 values for all the materials compared with GR-BxPC3 (Table S5). This is most likely due to the faster cell growth associated with C-BxPC3 (Figure 2). Monotherapy using cisPt-MSNs (9 or 21%wt) was only tested in GR-KCM and C-KCM cells showing the worst therapeutic performance (Table S5). For AsPC1 and HPAF-II cells, the monotherapy (Gem-MSN (10%wt)) reached the lowest IC50 values, 2514.2.3 ± 264.1 and 1927.3 ± 258.9 nM, respectively (Figure 5E,F and Table S6). Gem-cisPt-MSNs (10:9%wt) were the best combination with IC50 values of 5872.6 ± 275.2 and 5472.6 ± 306.8 nM.

Overall, the results from the cytotoxicity experiments indicate that monotherapy with Gem-MSN (10%wt) is the best approach for the treatment of the panel of PDAC cell lines. The combination therapy with Gem-cisPt-MSNs (10:9%wt) also shows a good therapeutic performance. As mentioned above, the difference in colloidal stability on these materials can have an impact on their cellular internalization, which could also affect their cytotoxicity. To test this hypothesis, the internalization of the Gem monotherapy (Gem-MSNs = 10% and 18%) and the combinations (Gem-cisPt-MSNs = 10:9, 18:9, and 18:21%wt) were evaluated in GR-BxPC3 and AsPC1 cells using flow cytometry (Figure S5). The uptake results in both cell lines clearly show that Gem-MSN (10%wt) and Gem-cisPt-MSNs (10:9%wt) have the highest values of internalization in GR-BxPC3 (>60%) and AsPC1 (>40%). On the contrary, the internalization values for Gem-MSN (18%wt) and Gem-cisPt-MSNs (18:9 and 18:2%wt) are much lower in GR-BxPC3 (<30%) and AsPC1 (<20%). Confocal microscopy confirmed the internalization of the MSNs materials in both GR-BxPC3 and AsPC1 cell lines, as evidenced by the green FITC labeled puncta observed in the micrographs (Figure S6).

The internalization results follow the same trend as the cytotoxicity data with both Gem-MSN (10%wt) and Gem-cisPt-MSNs (10:9%wt) showing the best performance. These results confirm the direct impact of the colloidal properties of the nanoparticles on their interaction with cancer cells and impact on internalization and cytotoxicity.

### 2.4 | MSN-Mediated Gem and/or cisPt Perturbed Cells in S Phases in Cell Cycle and Induced Apoptosis

**FIGURE 6 |:**
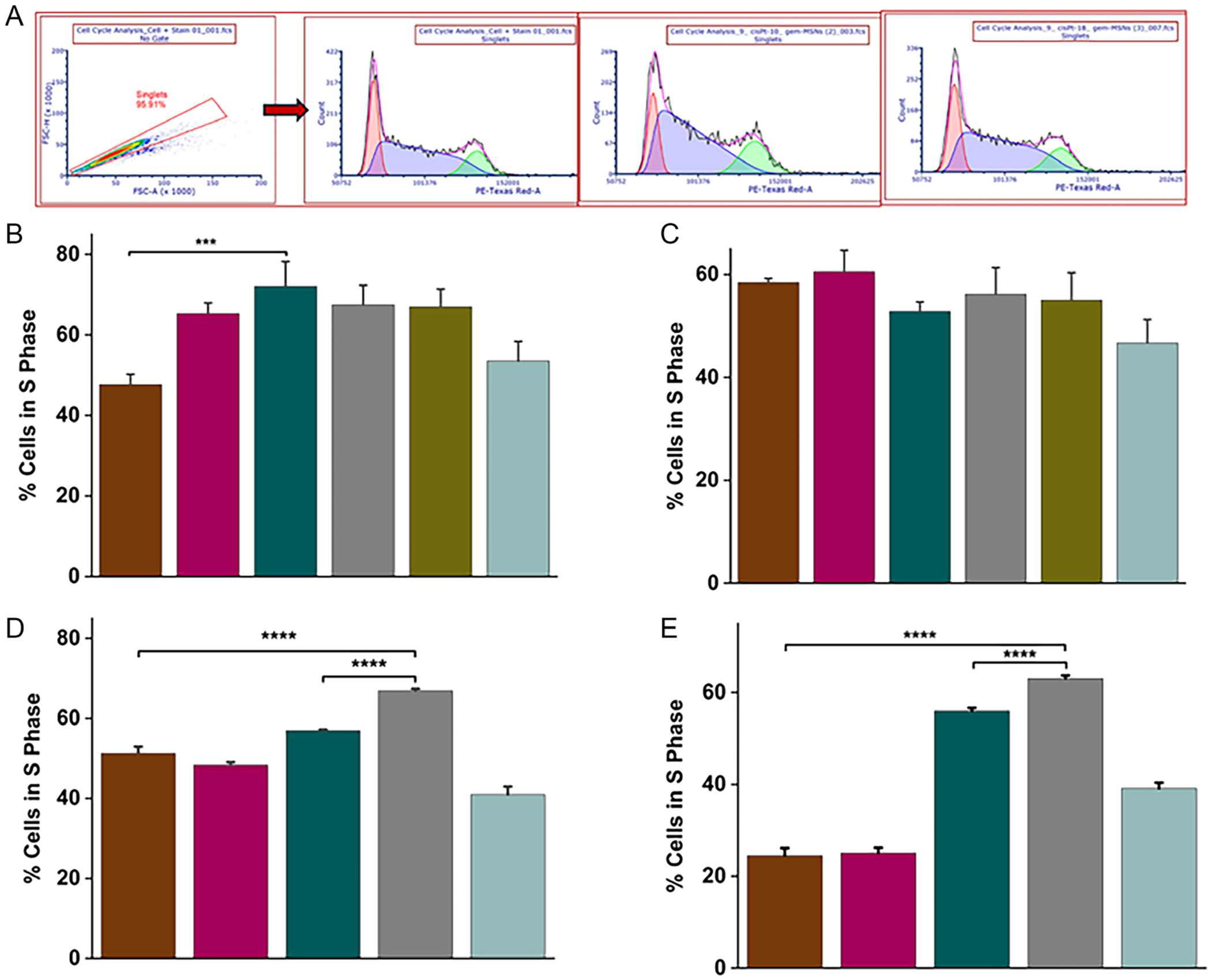
(A) Flow cytometry analysis was performed following treatment with various MSN formulations with subsequent PI staining for cell cycle analysis. Representative singlet gating was done followed by fitting the data for cell cycle in FCS Express 7 RUO (De Novo Software). (B) Percentage of cells in S phase associated with GR-KCM cells. (C) Percentage of cells in S phase associated with C-KCM cells. (D) Percentage of cells in S phase associated with GR-BxPC3 cells. (E) Percentage of cells in S phase associated with C-BxPC3 cells. Color code: Gem-MSN (10%wt) (brick red); Gem-MSN (18%wt) (burgundy); Gem-cisPt-MSN (10:9%wt) (dark green); Gem-cisPt-MSN (18:9%wt) (gray); Gem-cisPt-MSN (18:21%wt) (olive): control (blue-gray). All data are presented as mean ± SD from three independent experiments (*n* = 3). Statistical analysis was conducted using one-way ANOVA using Tukey’s multiple comparison test to assess differences between groups. Significance levels were denoted as follows: ****p ≤ 0.0001, ***p ≤ 0.001, **p ≤ 0.01, and *p ≤ 0.05.

Gem is a deoxycytidine analogue with a broad spectrum of cytotoxic activity. Gem exerts its activity primarily by inducing cell cycle arrest and cell death [50]. Previous reports have shown that Gem induced a time- and concentration-dependent cell cycle arrest at the S phase [45]. In addition, Gem also has relevant effects on cell apoptosis. cisPt damages DNA by inducing both inter- and intra-strand cross-links and its cytotoxic effects are conventionally considered to be agnostic of the cell cycle [51]. At a cellular level, cisPt generates heterogeneous responses with cells either dying or arresting. To investigate the effects of MSN-based therapies on cell cycle progression, we performed propidium iodide (PI)/RNase staining followed by flow cytometry to analyze DNA content in GR-KCM and GR-BxPC3 along with control cells (Figure 6A). This method enables quantification of cell populations in G0/G1, S, and G2/M phases, providing insights into DNA synthesis inhibition and mitotic arrest [52–54].

Across both GR-KCM and GR-BxPC3 cells, the MSN formulations consistently drove cells into S phase arrest, but the magnitude and context of this effect reveal important mechanistic distinctions. In GR-KCM cells, Gem-loaded MSNs alone were sufficient to disrupt cell cycle progression with Gem-MSNs (10 and 18%wt) showing an increase in the S phase compared with control cells 47.7 ± 2.5 versus 53.5 ± 4.9% and 65.3 ± 2.6% versus 53.5 ± 4.9%, respectively (Figure 6B). Co delivery with cisPt further amplified this effect, particularly Gem-cisPt-MSNs (10:9%wt) showing the highest increase in the S phase (72.1 ± 6.2% vs. 53.5 ± 4.9%). Although the three combination formulations produced statistically comparable S phase accumulation with Gem-cisPt-MSNs (18:9%wt) (67.5 ± 4.9%) and Gem-cisPt-MSNs (18:21%wt) (66.9 ± 4.4%), Gem-cisPt-MSNs (10:9%wt) stood out by coupling this arrest with the most pronounced depletion of G2/M cells, suggesting a more efficient blockade of DNA replication checkpoints rather than a generalized cytostatic response (Table S7). This pattern indicates that lower GEM loading paired with moderate cisPt may optimize the balance between replication stress and DNA damage signaling in this resistant background. In contrast, C-KCM cells exhibited a more uniform response: Gem-MSNs monotherapy and all Gem-cisPt-MSN combinations produced similar S phase enrichment (Figure 6C), while cisPt-MSNs remained ineffective (Tables S9 and S10). This convergence implies that in control cells, Gem-loaded MSN is the dominant driver of replication arrest, and the addition of cisPt does not substantially shift the cell cycle phenotype.

The human GR-BxPC3 model reinforced the theme that resistance heightens reliance on combination therapy. Gem monotherapy (10 and 18%wt) induced only modest S phase accumulation showing an increase in the S phase compared with control cells 51.3 ± 1.6 versus 40.9 ± 2.0% and 48.3 ± 0.7 versus 40.9 ± 2.0%, respectively (Figure 6D and Table S8). Whereas Gem-cisPt-MSNs (18:9%wt) produced a markedly stronger arrest (66.9 ± 0.5% vs. 40.9 ± 2.0%), accompanied by a clear reduction in G2/M cells (Table S11). Gem-cisPt-MSNs (10:9%wt) (56.9 ± 0.3%) showed a lower increase in the S phase, but still higher than the monotherapy. This suggests that in GR BxPC3, cisPt contributes more substantially to checkpoint disruption than in GR-KCM, potentially reflecting differences in DNA repair capacity or replication fork stability between the two resistant lines. Notably, increasing cisPt beyond this ratio was not pursued, as the KCM data indicated diminishing returns with higher cisPt loading. Monotherapy with cisPt-MSNs (9 and 21%wt) was not evaluated for GR-BxPC3 and C-BxPC3 because of the lack of impact on cell cycle arrest as shown with the GR/C-KCM cells. Finally, the C-BxPC3 cells diverged from the murine chemosensitive model: Gem-MSNs failed to induce S phase arrest monotherapy did not show an increase at the S phase (Figure 6E and Table S8); whereas the combinations restored this phenotype with Gem-cisPt-MSNs (18:9%wt) showing the highest increase in the S phase (63.0 ± 0.7% vs. 39.1 ± 1.3%). The other combination Gem-cisPt-MSNs (10:9%wt) (56.0 ± 0.7%) showed a lower increase in the S phase. This shift suggests that even in nominally C-BxPC3 cells, Gem-MSNs ability to trigger replication stress may be insufficient without concurrent DNA crosslinking, highlighting a species or lineage specific dependency on dual agent mechanisms.

Taken together, these patterns reveal that while Gem-loaded MSNs is the primary driver of S phase arrest in KCM and BxPC3 PDAC cell lines, particularly those with acquired resistance, derive greater benefit from coordinated Gem-cisPt delivery using MSNs. The consistent reduction in G2/M populations across responsive conditions further supports a model in which MSN-mediated codelivery enhances replication stress to a threshold that prevents cell cycle progression. These mechanistic differences highlight the value of ratiometric control in MSN formulations and point toward cell line specific optimization strategies for maximizing therapeutic impact.

### 2.5 | MSNs-Mediated Gem and/or cisPt Induced Apoptosis and ROS Generation

**FIGURE 7 |:**
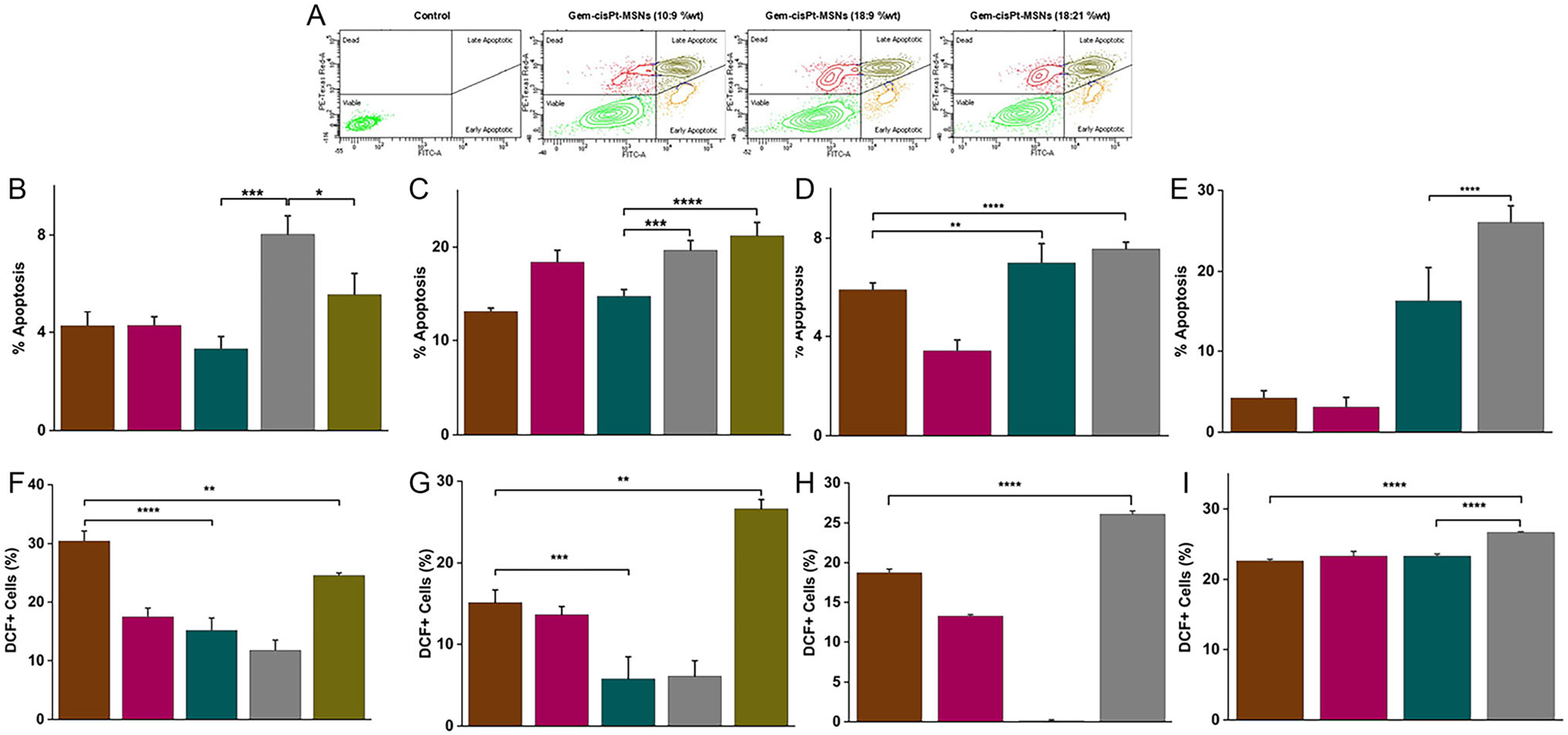
(A) Flow cytometry analysis was performed following treatment with various MSN formulations. Based on Annexin V-FITC and propidium iodide (PI) staining, cell populations were categorized as follows: Annexin V-FITC^−^/PI^−^ (FITC^−^/PI^−^) cells were considered viable; Annexin V-FITC^+^/PI^−^ (FITC^+^/PI^−^) cells were classified as early apoptotic; Annexin V-FITC^+^/PI^+^ (FITC^+^/PI^+^) cells were indicative of late apoptosis; and Annexin V-FITC^−^/PI^+^ (FITC^−^/PI^+^) cells were identified as dead. Percentage of apoptotic cells produce by the treatment of MSN materials for (B) GR-KCM, (C) C-KCM, (D) GR-BxPC3 and (E) C-BxPC3 cells. The percentage of DCF-positive (DCF^+^) cells was analyzed at 12h post-treatment. (F) GR-KCM cells, (G) C-KCM cells, H) GR-BxPC3 cells, and (I) C-BxPC3 cells. All data are presented as mean ± SD from three independent biological replicates (*n* = 3). Statistical analysis was conducted using one-way ANOVA using Tukey’s multiple comparison test to assess differences between groups. Significance levels were denoted as follows: ****p ≤ 0.0001, ***p ≤ 0.001, **p ≤ 0.01, and *p ≤ 0.05.

The apoptosis profiles across KCM and BxPC3 cells reveal clear distinctions between GR and chemosensitive phenotypes, as well as how each responds to single-agent versus combination MSN formulations. As expected for GR cell lines, both GR-KCM and GR-BxPC3 exhibited muted apoptotic responses to Gem-loaded MSNs, consistent with their reduced engagement of canonical Gem-induced death pathways (Figure 7B,D, and Table S9). In these resistant backgrounds, the addition of cisPt modestly but reproducibly enhanced apoptosis, indicating that dual-agent delivery can partially overcome the impaired apoptotic signaling characteristic of GEM resistance. Notably, Gem-cisPt-MSNs (18:9%wt) produced the most consistent improvement across both GR models, suggesting that this formulation achieves a more effective balance between replication stress and DNA damage to trigger cell death. The enhanced performance of the combination formulations in GR lines further highlights the therapeutic value of codelivery: while neither agent alone is sufficient to drive strong apoptosis in resistant cells, their coordinated release appears to amplify stress signals enough to partially restore apoptotic competence.

In contrast, C-KCM and C-BxPC3 cells displayed a broader dynamic range of apoptotic responses. C-KCM cells responded robustly to Gem-MSNs (10 and 18%wt) affording 13.1 ± 0.4% and 18.4 ± 1.3% of apoptotic cells, respectively. The combination formulations did not substantially exceed this effect with Gem-cisPt-MSNs (18:9 and 18:21%wt) inducing 19.7 ± 1.1 and 21.2% ± 1.4% (Figure 7C,E, and Table S13). These results imply that Gem-loaded MSNs alone are sufficient to activate apoptosis when intrinsic resistance mechanisms are absent. Meanwhile, C-BxPC3 cells showed a different pattern: Gem-MSNs alone induced minimal apoptosis, whereas the GEM–cisPt combinations produced a marked increase with Gem-cisPt-MSNs (10:9 and 18:9%wt) inducing high levels of apoptosis, 16.3 ± 4.1 and 26.1 ± 2.0%, respectively. This divergence between C-KCM and C-BxPC3 lines suggests lineage specific differences in how GEM engages apoptotic machinery.

Taken together, these findings highlight that apoptosis induction by MSN-based therapies is strongly shaped by both the intrinsic resistance status of the cells and the specific Gem:cisPt ratio. The Gem-cisPt-MSNs (18:9%wt) formulation emerges as the most broadly effective across resistant and sensitive models, supporting the concept that ratiometric control within MSNs can fine tune the balance between replication inhibition and DNA damage to optimize apoptotic outcomes.

Cellular reactive oxygen species (ROS) is critical mediator of apoptosis in response to chemotherapy [55, 56]. Both Gem and cisPt exert their cytotoxicity through ROS-dependent DNA and mitochondrial damage followed by externalization of phosphatidylserine on cell surface leading to programmed cell death [57, 58]. To investigate the ROS production, we performed flow cytometry using 2’ ,7’ -dichlorodihydrofluorescein diacetate (H_2_DCFDA) after treating GR-KCM and GR-BxPC3 along with various MSN formulations [59].

GR cells (GR-KCM and GR-BxPC3) displayed attenuated ROS generation in response to Gem monotherapy, with the exception of Gem-MSNs (10%wt) against GR-KCM, which afforded 30.4 ± 1.7% (Figure 7F,H, and Table S9). This is consistent with their reduced susceptibility to Gem induced oxidative stress. In these resistant lines, the addition of cisPt did not uniformly enhance ROS production; instead, ROS levels varied depending on the specific Gem:cisPt ratio. Notably, Gem-cisPt-MSNs (18:21%wt) formulation in GR-KCM and Gem-cisPt-MSNs (18:9%wt) formulation in GR-BxPC3 produced the strongest oxidative responses with 24.5 ± 0.5% and 26.1 ± 0.4% of ROS-positive cells, respectively. This suggests that only certain ratiometric combinations effectively amplify ROS to levels sufficient to engage apoptotic pathways in resistant cells. This pattern reinforces the idea that codelivery can partially bypass resistance mechanisms, but only when the balance between Gem driven replication stress and cisPt mediated DNA crosslinking is optimized.

In contrast, C-KCM and C-BxPC3 cells exhibited a different relationship between treatment and ROS generation. C-KCM cells showed modest ROS induction with Gem-MSN (Figure 7G and Table S9), and most Gem–cisPt-MSN combinations reduced ROS relative to monotherapy, except for Gem-cisPt-MSNs (18:21%wt) formulation that produces the highest levels of ROS, 26.6 ± 1.1%. This suggests that in C-KCM, Gem’s cytotoxicity is not strictly dependent on ROS amplification, and excessive cisPt loading may shift the mechanism toward oxidative damage rather than replication stress. Meanwhile, C-BxPC3 cells demonstrated relatively uniform ROS levels across monotherapy and most combination treatments, with only Gem-cisPt-MSNs (18:9%wt) affording slightly higher levels of ROS-positive cells, 26.7 ± 0.1 (Figure 7I and Table S9). This stability implies that in C-BxPC3 cells, ROS generation is not the primary determinant of treatment response, and apoptosis may instead be driven by checkpoint disruption or DNA damage signaling independent of oxidative stress.

Taken together, these findings highlight that ROS induction by MSN delivered Gem and cisPt is highly context dependent, shaped by both intrinsic resistance mechanisms and the specific drug ratios encapsulated within the nanoparticles. The formulations that most effectively induced apoptosis in GR-KCM and GR-BxPC3 cells, Gem-MSNs (10%wt) and Gem-cisPt-MSNs (18:9% wt), were also capable of generating meaningful ROS responses in these resistant backgrounds. This alignment supports a model in which ratiometric codelivery can recalibrate oxidative stress to overcome resistance, whereas in control cells, ROS plays a more variable and formulation dependent role. Overall, the ROS data reinforce the importance of precise drug ratio control within MSNs to tune oxidative and DNA damage pathways for maximal therapeutic benefit.

## 3 | Conclusions

In this work, we have developed murine (KCM) and human (BxPC3) GR cell lines. These cell lines show typical features of resistance associated with phenotypic plasticity, and various intracellular enzymatic and molecular adaptations. Characteristic biomarkers of EMT were analyzed, including Vimentin, E- and N-cadherin. In addition, various intracellular enzymatic and molecular adaptations associated with Gem resistance were studied such as human equilibrative nucleoside transporter 1 (hENT1), deoxycytidine kinase (dCK)s, and ribonucleotide reductase M1 subunit (RRM1). The observed upregulation of EMT-associated markers and altered expression of Gem-related proteins confirm the successful development of GR PDAC cell lines.

We synthesized MSN-based monotherapy and combination therapies with cisPt and Gem. Different amounts and ratios were loaded to MSNs including cisPt-MSNs (9 and 21%wt), Gem-MSNs (10 and 18%wt), and Gem-cisPt-MSNs (Gem: cisPt = 10:21, 10:9 and 18:9%wt). The cytotoxic impact of these materials was determined using the MTS, which showed that Gem-MSN (10%wt) and Gem-cisPt-MSNs (10:9%wt) exhibited the strongest cytotoxicity in GR PDAC cell lines. This can be explained by a higher nanoparticle internalization in GR cells due to better colloidal stability. To have a better understanding on the cell death mechanisms associated with the MSN platforms, we studied the cell cycle arrest and apoptosis. Interestingly, these assays demonstrated that Gem-cisPt-MSNs (18:9%wt) exhibited the highest apoptotic effect and enhanced cell-cycle arrest in GR PDAC models. The difference in the performance of MSN materials in cytotoxicity, cell cycle arrest, apoptosis, and ROS generation is most likely because the combined Gem-cisPt-MSNs are effective cytostatic agent, by stopping the cell cycle and priming the cells to death through apoptosis, but the overall effect is not enough to cause the cytotoxic effect detected by the MTS assay. In that case, monotherapy using Gem-MSN (10%wt) has a better cytotoxic effect.

Overall, we have shown that MSN is a robust platform to develop mono and combination chemotherapies for the effective treatment of GR PDAC cell lines. We envision that this system can be tested in preclinical settings using in vivo PDAC models.

## 4 | Experimental Section

### 4.1 | Synthesis of Gemcitabine and Cisplatin Coloaded MSNs with Tunable Drug Ratios

MSNs were synthesized using a sol–gel method and cocondensed with 3-aminopropyltriethoxysilane to introduce surface amine groups, yielding aminopropyl-functionalized MSNs. CisPt-conjugated MSNs (cisPt-MSNs) with defined weight loadings were synthesized by reacting amine-functionalized MSNs with cisPt prodrug in the presence of EDC in DMSO. For 9% cisPt loading, 62 mg of MSNs was dispersed in 1.24 mL of DMSO containing 13.02 μL of triethylamine (TEA), followed by the addition of a solution containing 24.8 mg of cisPt prodrug and 44.64 mg of EDC in 1.24 mL of DMSO. For 21% cisPt loading, 705 mg of AP-MSNs was dispersed in 21.15 mL of DMSO with 148.05 μL TEA, and then treated with a 14.1 mL DMSO solution containing 282 mg of cisPt prodrug and 507.6 mg of EDC. Both reactions were stirred at room temperature for 24 h. The nanoparticles were collected by centrifugation, washed thoroughly with ethanol, and stored. The platinum content was determined via AAS using the combined supernatants from the reaction and wash steps. To introduce phosphonate groups, MSNs or cisPt-MSNs (200 mg) were dispersed in 13 mL of nanopure water, and 113.5 μL (0.2 mmol) of trimethylphosphite was added. The pH was adjusted to 6–7, and the suspension was stirred at 40°C for 3 h. The resulting phosphonate-modified nanoparticles (Phos-MSNs or Phos-cisPt-MSNs) were recovered by centrifugation and washed with ethanol. Subsequently, a PEI (MW 1.8 kDa) coating was applied to enhance colloidal stability and facilitate further conjugation. Phos-MSNs or Phos-cisPt-MSNs (100 mg) were dispersed in 40 mL of ethanol, and 10 mL of PEI solution (2.5 mg/mL in ethanol) was added. The mixture was stirred for 1 h at room temperature, followed by centrifugation and triple washing with ethanol to yield PEI-MSNs or PEI-cisPt-MSNs. The amount of PEI grafted and the free amines onto the surface were quantified by ninhydrin assay, wherein 1 mg of PEI-coated MSNs was reacted with 1 mL of ninhydrin reagent (15 mg/mL in ethanol) for 24 h, and absorbance was measured at 575 nm using UV–vis spectroscopy against a PEI calibration curve. Gem conjugation was carried out in a two-step process. First, PEI-MSNs or PEI-cisPt-MSNs (30 mg) were dispersed in 15 mL of anhydrous acetonitrile and reacted with SPDP (15–22 mg, 48 μmol depending on the desired % loading of Gem) under stirring at room temperature for 24–72 h to form SPDP-MSNs or SPDP-cisPt-MSNs. SPDP conjugation efficiency (up to 86%) was quantified by ninhydrin assay. In the second step, Gem prodrug was conjugated via disulfide exchange. SPDP-functionalized nanoparticles were dispersed in 10 mL of methanol, and a solution of Gem prodrug (30 mg, 85.4 μmol in 5 mL methanol) was added. The reaction was stirred for 72 h, and the resulting Gem-MSNs or Gem-cisPt-MSNs were purified by centrifugation and sequential washing with methanol and ethanol. To generate formulations with specific dual drug loadings, the following strategies were used. For Gem-cisPt-MSNs (10:9%wt), 30 mg of SPDP-cisPt-MSNs was reacted with 30 mg of Gem prodrug for 72 h. For Gem-cisPt-MSNs (18:9%wt) and Gem-cisPt-MSNs (18:21%wt), either an additional 20–25 mg of Gem prodrug was added to the initial mixture and stirred for another 72 h, or the same amount of Gem prodrug (30 mg) was allowed to react for an extended period (up to 6 days) to increase the loading. Control formulations containing Gem-MSNs (10 or 18%wt) were synthesized following the same conjugation protocol using SPDP-MSNs, adjusting the amount of Gem prodrug or reaction time accordingly.

### 4.2 | Cell Culture Conditions

KCM cells were thawed from cryogenic condition and cultured in DMEM media supplemented with 10% FBS, 1% penicillin and streptomycin, 1% Glutamax, and 1% NEAA. BxPC3 cells were cultured in RPMI media supplemented with 10% FBS and 1% penicillin. AsPC1 cells were also cultured in RPMI media supplemented with 10% FBS and 1% penicillin. HPAFII cells were required in EMEM media supplemented with 10% FBS, 1% penicillin, 1% Glutamax and 1% NEAA.

### 4.3 | Developing Gemcitabine Resistant KCM and BxPC3 Cell Lines

KCM cells are derived from a PDAC mouse model that spontaneously develops pancreatic adenocarcinoma, and these cells express human MUC1 [60, 61]. KCM cells were thawed from cryogenic condition and cultured in DMEM media supplemented with 10% FBS, 1% penicillin and streptomycin, 1% Glutamax and 1% NEAA, passaged for seven times before separating those cells in two T75 culture flasks. One flask was kept as control flask (C-KCM), and the second flask was treated with a specific dose of Gem as described below. First, both Gem-treated flasks were seeded with 5.0 × 10^5^ cells and allowed to reach ≈80%–90% confluency. The treated cells were exposed to equivalent doses of IC50 dose Gem (11.80 ± 2.44 nM) for 48 h and cells were allowed to grow to confluency for at least 24 h before the following passage. Cells were treated under the same conditions for 12 passages and kept without Gem treatment for the next two passages. The Gem treatment was then resumed at a dose 10 times the IC_50_. This iteration continued for 31 passages for KCM control and 36 passages for Gem treated KCM cells. Finally, the viability or cell proliferation study of both C-KCM and GR-KCM cells were evaluated using the MTS assay. Both cells were exposed to 1–1000 nM of Gem and the median lethal dose for both cell lines were determined (Table S1). After calculating the fold resistance, the GR-KCM cells were almost 9 times more resistant to Gem compared to control cells. The median lethal doses of GR-KCM and C-KCM cells were 61.50 ± 6.40 nM and 6.90 ± 1.90 nM (Table S1). All the cells were maintained at 37°C in the incubator with 5% CO2.

To develop GR-BxPC3 a similar protocol was followed, but Gem concentration was kept at equivalent dose of IC_50_ due to sensitivity of these human PDAC cells derived to Gem. After 19 passages the GR-BxPC3 showed almost three times more resistance to C-BxPC3 cells. The IC_50_ of Gem resistant and control cells were 166.90 ± 25.56 nM and 56.50 ± 7.71, respectively, with a fold resistance of 2.95 (Table S1).

### 4.4 | Protein Extraction and Western Blotting KCM and BxPC3 Cells

Protein lysates from C-BxPC3/C-KCM and GR-BxPC3/GR-KCM cells were prepared using 5 × 10^6^ to 1 × 10^7^ cells. Cells were washed once with cold PBS and lysed with Pierce radioimmuno-precipitation assay (RIPA) lysis and extraction buffer and a Halt Protease and Phosphatase Inhibitor Cocktail, EDTA-free at a 99:1 ratio. For every 1 × 10^7^ cells placed in the centrifuge tube, 1 mL of lysis buffer was added. The lysate was incubated on ice for 10 min and then centrifuged at 14,000 rpm for 15 min. The supernatant was collected, and total protein concentration was determined using Pierce Dilution-Free Rapid Gold bicinchoninic acid Protein Assay Kit, following the manufacturer’s protocol.

To measure the target proteins using Western blot, 20–25 μg of sample was mixed at a 3:1 ratio with loading dye, consisting of Laemmli sample buffer and 2- mercaptoethanol. Sample mixture was boiled at 90°C for 5 min then centrifuged at 14,500 rpm for 5 min and run on a sodium dodecyl sulfate-polyacrylamide gel electrophoresis (SDS-PAGE) with 1X Tris/Glycine/SDS buffer diluted using nanopure water at 200V for 25–30 min. The proteins were then transferred onto an Immun-blot polyvinylidene difluoride membrane with cold 1X Tris/Glycine buffer with methanol and run at 100V for 1 h at 4°C. The membrane was washed using 1X Tris-buffered saline with Tween 20 (1X TTBS) and blocked with 5% blotto milk at room temperature. The membrane was then incubated in the primary antibodies diluted by volume with blocking agent overnight 4°C with agitation. The membrane was then incubated in a secondary antibody of anti-rabbit IgG or antimouse IgG diluted in blocking agent for 1 h at room temperature. The membrane was then washed with 1X TTBS and developed using Clarity Max Western enhanced chemiluminescence substrates. Protein bands were visualized and imaged using the ChemiDoc Imaging System from Bio-Rad.

### 4.5 | Confocal Microscopy for EMT Characterization

C/GR-BxPC3 and C/GR-KCM cells were seeded on 6-well plates with coverslips with a cell density of 2 × 10^5^ cells and incubated for 24 h to allow cells to adhere to coverslips. After 24 h, wells were washed with PBS, fixed with 5% formalin and blocked with a buffer of 1X PBS, 5% normal goat serum and 0.3% Triton X-100. Primary antibodies were diluted in antibody buffer consisting of 0.3% Triton X-100 and 1% Bovine Serum Albumin (BSA) in PBS and incubated with cells overnight at 4°C with agitation. Following PBS washes, Alexa Fluor 568-conjugated secondary antibody goat antirabbit IgG was applied for 2 h at room temperature in the dark. Nuclei were stained with NucBlue Fixed Cell Ready Probes Reagent (DAPI) for 3–5 min, followed by washes with nanopure water and PBS. Coverslips were mounted using ProLong Gold Antifade Mountant onto glass slides, and samples were imaged with a Leica Stellaris 8 confocal microscope. Details of image analysis are provided in supplemental information.

### 4.6 | RNA Isolation and Quantitative Real-Time PCR (qRT-PCR)

The Monarch Total RNA Miniprep Kit (New England Biolabs) was used to extract total RNA from 1 × 10^6^ BxPC3 or KCM cells following the manufacturer’s protocol. The RNA samples were treated with an on-column DNase I (Invitrogen) to remove any possible contamination from genomic DNA. Isolated RNA samples were quantified using a Nanodrop One Spectrophotometer with WiFi and Qubit 4 Fluorometer (ThermoFisher) and stored at −20°C.

Quantitative RT-PCR was performed on a QuantStudio 3 (Applied Biosystems). The Power SYBR Green RNA-to-CT 1-step kit (Applied BioSystems) was used to perform the qRT-PCR according to the manufacturer’s protocol. Briefly, the RT–PCR mixture consisted of 10 μL Power SYBR Green RT-PCT Mix (2×), 0.16 μL RT Enzyme Mix (125×), 8 μL of RNA template (10 ng), variable volume of forward and reverse primer (0.2 or 0.3 M final concentration), and RNase-free water to make the final reaction volume of 20 μL. The list of primers is provided in the supplemental information (S22). The cycling profile for all the reactions was 1 cycle of 48°C for 30 min, 95°C for 10 min, 40 cycles of 95°C for 15 s, and 60°C for 15 s followed by a melt curve.

The data obtained from the qRT-PCR were analyzed using information on the cycle number (Ct values). The sample that expresses more of a given gene product has a lower Ct value while the lower expressing sample has a higher Ct value. Relative quantification of mRNA levels has been determined through the use of the relative fold change calculation according to Pfaffl (2001) [62].

### 4.7 | Comparative IC_50_ Profiling

For the MTS assays, C-KCM/C-BxPC3, GR-KCM/GR-BxPC3, AsPC-1, and HPAF-II were seeded into 96-well plates at densities of 5× 10^2^, 4 × 10^3^ and 2×10^3^ 500, 4,000, 2,000 and 2000 cells per well, respectively, and incubated for 24 h to allow cell adherence. Subsequently, the cells were treated with varying concentrations of chemotherapeutic agents: C-KCM/GR-KCM cells received Gem and cis-Pt in combination at concentrations ranging from 0.1 to 100 μg/mL for 48 h. C-BxPC3/GR-BxPC3 cells were exposed to cisPt monotherapy at 0.1–350 μg/mL, Gem monotherapy at 0.1–100 μg/mL, and combination treatments within the same concentration range. AsPC-1 and HPAF-II cells were treated with drug concentrations ranging from 0.1 to 70 μg/mL. Following treatment, cells were washed once with PBS, replenished with fresh media, and incubated for an additional 24 h to facilitate recovery. Cell viability was then assessed using the CellTiter 96 AQueous One Solution Cell Proliferation Assay (Promega), which utilizes the MTS tetrazolium compound. In this assay, 20 μL of the MTS reagent was added to each well containing 100 μL of media, and plates were incubated at 37°C for 2.5–3.5 h, depending on the cell line. The resulting absorbance was measured at 490 nm using a Multiskan FC plate reader, providing a quantitative measure of viable, metabolically active cells.

### 4.8 | Cell Cycle Analysis

C-KCM/GR-KCM cells were seeded at a density of 1.1 × 10^5^ and allowed to grow for 24 h. For all BxPC3 subtypes, the seeding density was 6.0 × 10^5^. Subsequently, cells were exposed to Gem- and/or cisPt-loaded MSNs, as well as positive control, at concentrations of 10 μg/mL for KCM and 120 μg/mL for BxPC3 for 24 h. Then the cells were harvested using trypsin, washed once with ice cold PBS and then the cell pellets were resuspended in 0.5 mL of cold PBS before fixing with 4.5 ml of 70% prechilled ethanol. The fixed cells were kept on ice for 30 min followed by overnight freezing at −20°C. The cells were washed once with ice cold PBS and then the cells were suspended in 0.5 mL of FxCycle PI/RNase staining solution and incubated at room temperature for 15–30 mins followed by flow cytometry analysis. The data were presented as the percentage of cells that were arrested in the G1/S/G2-M cycle, and the results were reported as mean ± SD of three independent experiments (*n* = 3).

### 4.9 | Analysis of Apoptosis

C-KCM/GR-KCM cells were seeded in 6-well plates at 7.0 × 10^4^ cell density and incubated for 24 h. For all BxPC3 subtypes, the seeding density was 6.0 × 10^5^. All cells were then treated with therapeutic Gem- and/or cisPt-loaded MSNs, together with PEI-MSNs as a positive control, for 24 h using the same concentrations as in the previous experiment. Supernatants were collected to recover apoptotic cells, after which adherent cells were trypsinized and harvested by centrifugation. Cells were washed once with ice cold PBS before resuspending the cell pellets in 100 μL of 1X binding buffer. Cells were then stained with 2 μLof Annexin V-FITC solution followed by 5 μL of propidium iodide staining for 20 min at room temperature in the dark. 400 μLof 1X binding buffer was added to all tubes before analyzing the samples using flow cytometry (BD LSRFortessa). The flow cytometry data was analyzed using BD FACSDiva 8.0.1. Viable cells were negative for both FITC and PI (FITC^−^ and PI^−^); early apoptotic cells were FITC positive and PI negative (FITC^+^ and PI^−^); late apoptotic cells were positive for both stains (FITC^+^ and PI^+^); and dead cells appeared negative for FITC and positive for PI (FITC^−^ and PI^+^). Percentage of cells that were apoptotic were presented with both early plus late apoptotic cells and the results were presented as mean ± SD of three independent experiments (*n* = 3).

### 4.10 | Statistical Analysis

All of the statistical analysis was conducted using one-way ANOVA using Tukey’s multiple comparison test to assess differences between groups. The sample size, n has been mentioned at each of the figure legends and significance levels were denoted as follows: ****p ≤ 0.0001, ***p ≤ 0.001, **p ≤ 0.01, *p ≤ 0.05, and not significant (ns) for *p* > 0.05.

## Supplementary Material

ESI

Additional supporting information can be found online in the Supporting Information section. **Supporting Scheme S1**: Chemical synthesis of the cisPt prodrug. **Supporting Scheme S2**: Chemical synthesis of the Gem prodrug. **Supporting Fig. S1**: Expression of EMT markers in KCM and BxPC3 cells. Quantification of protein levels of Vimentin, N- and E0cadherin in C/GR-KCM and C/GR-BxPC3 cells (left) and EMT marker expression in C-KCM and C-BxPC3 cells. Statistical comparisons were conducted using one-way ANOVA followed by Tukey’s multiple comparison test. Significance levels are denoted as follows: **p ≤ 0.01, and *p ≤ 0.05. **Supporting Fig. S2**: Nitrogen adsorption-desorption isotherms and pore size distribution of mesoporous silica nanoparticles (MSNs). **Supporting Fig. S3**: Evaluation of long-term colloidal stability (Cell media) of the formulations through Z-average hydrodynamic diameter and PDI-tracking over time, demonstrating the stability of nanoparticles under storage or physiological conditions. Color code: AP-MSNs (Cyan); cisPt-MSN (Lime Green); Phos-cisPt-MSN (Burnt Orange); PEI-cisPt-MSN (Navy Blue); SPDP-cisPt-MSN (Lavender); Gem-MSN (10 %wt) (Brick Red); Gem-MSN (18 %wt) (Burgundy); Gem-cisPt-MSN (10:9 %wt) (Dark Green); Gem-cisPt-MSN (18:9 %wt) (Gray); Gem-cisPt-MSN (18:21 %wt) (Olive). **Supporting Fig. S4**: Dose-response curves of mono- and dual-drug loaded MSNs across pancreatic cancer cell lines. (A) GR-KCM, (B) C-KCM, (C) GR-BxPC3, (D) C-BxPC3, (E) AsPC1 and (F) HPAF-II. Error bars represent the standard deviation of three biological replicates (*n* = 3). Color code: AP-MSNs (Cyan); cisPt-MSN (Lime Green); Phos-cisPt-MSN (Burnt Orange); PEI-cisPt-MSN (Navy Blue); SPDP-cisPt-MSN (Lavender); Gem-MSN (10 %wt) (Brick Red); Gem-MSN (18 %wt) (Burgundy); Gem-cisPt-MSN (10:9 %wt) (Dark Green); Gem-cisPt-MSN (18:9 %wt) (Gray); Gem-cisPt-MSN (18:21 %wt) (Olive). **Supporting Fig. S5**: Internalization of Gem monotherapy (Gem-MSNs = 10 and 18 %wt) and the combinations (Gem-cisPt-MSNs = 10:9, 18:9 and 18:21 %wt) were evaluated in GR-BxPC3 (left) and AsPC1 (right) cells using flow cytometry. Gem-MSN (10 %wt) (Brick Red); Gem-MSN (18 %wt) (Burgundy); Gem-cisPt-MSN (10:9 %wt) (Dark Green); Gem-cisPt-MSN (18:9 %wt) (Gray); Gem-cisPt-MSN (18:21 %wt) (Olive). All data are presented as mean ± standard deviation (SD) from three independent biological replicates (*n* = 3). **Supporting Fig. S6**: Live-cell imaging with internalization of MSNs in GR-BxPC3 and AsPC1 cells at 40x magnification. Nuclei-stained blue using NucBlue and MSNs-FITC conjugated stained as green. **Supporting Fig. S7**: Flow cytometry analysis was performed following treatment with various mesoporous silica nano-particle (MSN) formulations. Based on Annexin V-FITC and propidium iodide (PI) staining, cell populations were categorized as follows: Annexin V-FITC^−^/PI^−^ (FITC^−^/PI^−^) cells were considered viable; Annexin V-FITC^+^/PI^−^ (FITC^+^/PI^−^) cells were classified as early apoptotic; Annexin V-FITC^+^/PI^+^ (FITC^+^/PI^+^) cells were indicative of late apoptosis; and Annexin V-FITC^−^/PI^+^ (FITC^−^/PI^+^) cells were identified as dead. Data represents the mean ± SD of three independent experiments (*n* = 3). **Supporting Table S1**: IC_50_ values of free Gem for KCM and BxPC3 cells. **Supporting Table S2**: Drug content in various ratios of Gem-cisPt-MSNs (%wt). **Supporting Table S3**: Pore-surface Analysis Data of nanomaterials (BET-BJH method). **Supporting Table S4**: Dynamic Light Scattering Analysis Data of nanomaterials. **Supporting Table S5**: IC_50_ values in various ratios of Gem-cisPt-MSNs (KCM and BxPC3 Cell lines). **Supporting Table S6**: IC_50_ values in various ratios of Gem-cisPt-MSNs (AsPC1 and HPAFII Cell lines). **Supporting Table S7**: Cell cycle analysis for C-KCM and GR-KCM cell subtype after cisPt and/or Gem-MSNs treatment. **Supporting Table S8**: Cell cycle analysis for C-BxPC3 and GR-BxPC3 cell subtype after cisPt and/or Gem-MSNs treatment. **Supporting Table S9**: Percentage of apoptotic KCM and BxPC3 cells after cisPt and/or Gem-MSNs treatment. **Supporting Table S10**: Time-dependent intracellular ROS generation induced by Gem- and/or cisPt-loaded MSNs. (A) GR-KCM cells, (B) C-KCM cells, (C) GR-BxPC3 cells, and (D) C-BxPC3 cells.

## Data Availability

The data that supports the findings of this study are available in the supplementary material of this article.
