# Supplementary material for: Mesoporous Silica Nanoparticles With Customized Drug Ratio/Loading for Effective Treatment of Gemcitabine-Resistant Pancreatic Tumors
Source: Adv Nanobiomed Res. Author manuscript; Available in PMC 2026 May 17. (PMC13179672; doi:10.1002/anbr.202500255)
Supplement: ESI [file NIHMS2168781-supplement-ESI.pdf]

Mesoporous Silica Nanoparticles with Customized Drug Ratio/Loading for Effective Treatment of Gemcitabine-Resistant Pancreatic Tumors

Tamanna Binte Huq[#1,2], Sudip Kumar Dam[#1,2], Doaha Awad[#3], Punnya Anil Kumar Jeeja[1,2], Farzana Ferdous[3], Juan L. Vivero-Escoto[1,2,4*]

1 – Department of Chemistry, University of North Carolina at Charlotte, Charlotte, NC 28223, USA.
2 – Chemistry and Nanoscale Science Program, University of North Carolina at Charlotte, Charlotte, NC 28223, USA.
3 – Department of Biological Sciences, University of North Carolina at Charlotte, Charlotte, NC 28223, USA.
4 – Center for Biomedical Engineering and Science, University of North Carolina at Charlotte, Charlotte, NC 28223, USA.

[#]These authors contributed equally.

S1. Materials and Methods

Materials and Reagents

Hexadecyltrimethylammonium bromide (CTAB), tetraethyl orthosilicate (TEOS), (3-aminopropyl)triethoxysilane (APTES), diethanolamine (DEA), 3-(trihydroxysilyl)propyl methylphosphonate monosodium salt (TPMP), poly(ethyleneimine) polymer (PEI; MW = 1.8 kDa), N-succinimidyl-3-(2-pyridyldithio)propionate (SPDP), gemcitabine hydrochloride (Gem), cis-diamineplatinum dichloride (cisplatin/cisPt), hydrogen peroxide (H_2O_2), O-(benzotriazol-1-yl)-N,N,N',N'-tetramethyluronium tetrafluoroborate (TBTU), N,N-diisopropylethylamine (DIPEA), 1-ethyl-3-(3-dimethylaminopropyl)carbodiimide (EDC), trifluoroacetic acid (TFA), succinic anhydride, S-trityl-mercaptopropionic acid, triethylsilane (TEA), and ninhydrin reagent were procured from Sigma Aldrich. Roswell Park Memorial Institute (RPMI 1640), fetal bovine serum (FBS), Dulbecco's Modified Eagle Medium (DMEM), penicillin-streptomycin, phosphate-buffered saline (PBS, 1X), and trypsin were purchased from Corning. Glutamax was obtained from Gibco, while non-essential amino acids (NEAA) were sourced from Quality Biologicals. Additional fetal bovine serum (FBS) was purchased from Atlanta Biologicals. The CellTiter 96® AQueous Assay was acquired from Promega (Madison, WI, USA), the BD Pharmingen™ Annexin V-FITC Apoptosis Detection Kit was obtained from BD Biosciences, and FxCycle™ PI/RNase Staining Solution was purchased from Thermo Fisher Scientific, USA. The Apoptosis Detection Kit (ab206386) was purchased from Abcam Co. LTD (USA). For lysis of cells Pierce radioimmunoprecipitation assay (RIPA) lysis and extraction buffer, Halt Protease and Phosphatase Inhibitor Cocktail, EDTA, Pierce Dilution-Free Rapid Gold bicinchoninic acid (BCA) Protein Assay Kit were obtained from ThermoFisher Scientific. The primary antibodies for Western blot and confocal microscopy were β-actin, RRM1, Vimentin, E-cadherin, N-cadherin were purchased from Cell Signaling Technology and dCK and hENT1 were sourced from Santa Cruz Biotechnology. The secondary antibodies were mouse-anti-IgG HRP and rabbit-anti-IgG HRP were obtained from Cell Signaling Technology for Western blot and Alexa Fluor 568-conjugated secondary antibody goat anti-rabbit IgG from ThermoFisher Scientific for

confocal microscopy. NucBlue™ Fixed Cell ReadyProbes™ Reagent and NucBlue™ Live Cell ReadyProbes™ Reagent were obtained from Thermo Fisher Scientific were used for confocal microscopy. The Monarch Total RNA Miniprep Kit from New England Biolabs was used to extract total RNA and treated with an on-column DNase I from Invitrogen to remove any possible contamination from genomic DNA. The Power SYBR Green RNA-to-CT 1-step kit from Applied BioSystems was used to perform the qRT-PCR.

A Malvern Instrument Zetasizer Nano (red laser 633 nm) (Malvern Instrument Ltd., Malvern, UK) was utilized for hydrodynamic size and zeta potential measurements. Surface area and pore size analyses of MSNs were conducted using the NOVA 2200e Quantachrome surface area and pore analyzer. A Thermo Nanodrop 1000 spectrophotometer was employed for gemcitabine release studies and protein A280 detection. Atomic absorption spectroscopy (AAS) has been used for quantifying cisPt loading. Cell viability experiments were conducted using a Multiskan FC plate reader from Fisher Scientific, while flow cytometry experiments were performed using a BD LSRFortessa. Imaging for Western blot was done using ChemiDoc Imaging System from BioRad and confocal microscopy imaging was done using the Stellaris 8 confocal microscope from Leica Microsystems. For qPCR, QuantStudio 3 by ThermoFisher Scientific was used. Isolated RNA samples were quantified using a Nanodrop One Spectrophotometer with WiFi and Qubit 4 Fluorometer from ThermoFisher.

S2. Approaches for the Synthesis and Characterization of Nanomaterials

S2.1 Synthesis of Cisplatin Prodrug

The synthesis of cisPt prodrug involves a two-step process adapted from established methodologies with slight modifications to optimize the procedure.[1] The initial step involves oxidizing cisPt to dihydroxycisplatin. cisPt (200 mg, 0.67 mmol) was dissolved in nanopure water (9 mL, pH 7), and hydrogen peroxide (30 %wt, 1 mL) was added. The reaction mixture was stirred at 70°C for 5 h under a nitrogen atmosphere. The mixture was then cooled to room temperature and stirred overnight. The product was washed with ice-cold water and ethanol. As the second step, dihydroxycisplatin (100 mg, 0.3 mmol) was dissolved in 4 mL of dimethyl sulfoxide (DMSO), followed by the addition of succinic anhydride (120.4 mg, 1.2 mmol). The reaction mixture was stirred at 70 °C for 24 h in the dark. The product was vacuum-dried, washed with cold acetone, and collected as disuccinotocisplatin.

Characterization: ^{1}H NMR (300 MHz, DMSO-d6): δ 2.61-2.77 (m, 2H), 2.21-2.32 (m, 2H).^{13}C NMR (300 MHz, DMSO-d6): δ 180.1, 174.3, 30.9, 29.1. IR Spectroscopy: Step 1 Yield: 45 wt%. FT-IR (cm^{-1}): 3517 (O-H), 3267 (N-H). Step 2 Yield: 65 %wt. FT-IR (cm^{-1}): 3457 (O-H), 3262 (N-H), 2920 (C-H), 1705 (C=O).

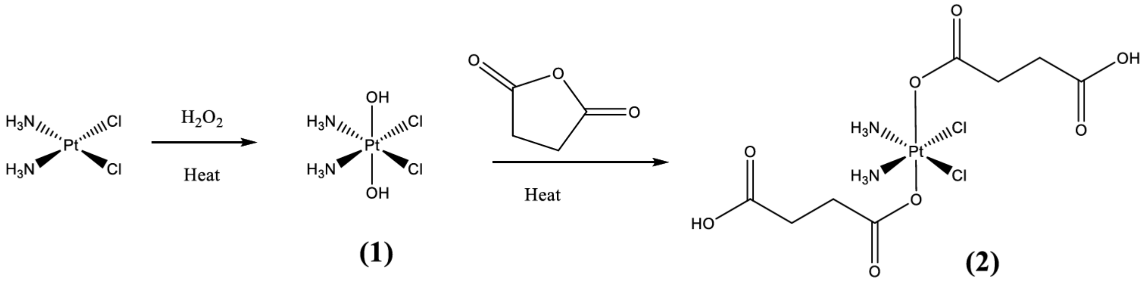

(1) (2)

Scheme 1: Chemical synthesis of the cisPt prodrug.

S2.2 Synthesis of Gemcitabine Prodrug

The gemcitabine (Gem) prodrug synthesis was carried out in two steps based on literature protocols with modifications.[1]
S-trityl-mercaptopropionic acid (136 mg, 0.4 mmol) and TBTU (143 mg, 0.44 mmol) were dissolved in anhydrous DMF (2 mL), followed by the addition of gemcitabine hydrochloride (200 mg, 0.64 mmol). DIPEA (244 µL, 1.58 mmol) was added, and the reaction stirred at room temperature for 72 h. The product was precipitated with cold brine, filtered, and dried under reduced pressure. After drying, one time wash with nanopure water confirms the removal of excess salt from the step 1 product. Yield: 76 %wt.
Characterization: ^{1}H NMR (300 MHz, MeOD-d4): δ 7.78 (d, 1H, J = 3.6 Hz), 7.21-7.23 (m, 16H), 6.25-6.28 (t, 1H, J = 8.6 Hz), 4.89-5.55 (d, 1H, J = 10.8 Hz), 4.51-4.67 (m, 1H), 4.88-4.95 (m, 2H), 4.20-4.25 (m, 1H), 2.11-2.23 (m, 2H), 2.45-2.89 (m, 2H). ^{13}C NMR (300 MHz, MeOD-d4): δ 177.43, 156.76, 176.31, 142.65, 134.28, 127.67, 126.45, 92.31, 87.94, 83.15, 70.42, 67.89, 59.21, 37.76, 27.03. FT-IR (cm^{-1}): 3320 (O–H), 2950 (C–H), 1735 (C=O), 1640 (C=C), 1570 (C=N), 1140 (C–O).
3-Tritylthio-gemcitabine (80 mg, 0.14 mmol) was dissolved in a 1:1 mixture of TFA and DCM (2 mL). Triethylsilane (0.5 mL, 3.0 mmol) was added, and the mixture stirred at room temperature for 1 h. The product was dried with Rotavap, sonicated and vortexed with diethyl ether (5×5 mL), and dried again to yield Gem prodrug. Yield: 18 %wt.
Characterization: ^{1}H NMR (300 MHz, MeOD-d4): δ 8.32 (d, 1H, *J* = 6.6 Hz), 7.48–7.42 (t, 1H, *J* = 7.5 Hz), 6.26–6.20 (t, 1H, *J* = 7.2 Hz), 4.28–4.22 (m, 1H), 3.95–3.91 (m, 2H), 2.77–2.72 (m, 4H). ^{13}C NMR (300 MHz, MeOD-d4): δ 172.48, 144.69, 128.97, 96.85, 85.33, 61.92, 58.44, 37.72. FT-IR (cm^{-1}): 3265 (O–H), 2940 (C–H), 1725 (C=O), 1645 (C=C), 1560 (C=N), 1142 (C–O).

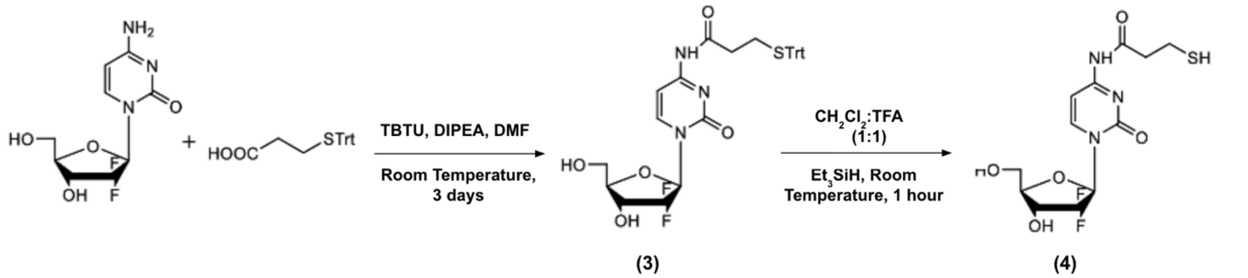

Scheme 2: Chemical synthesis of the Gem prodrug.

S3. Synthesis of Gem-cisPt-MSNs

S3.1 Synthesis of AP-MSNs (Amino-Propylated Mesoporous Silica Nanoparticles)

AP-MSNs were synthesized using a modified protocol based on previously established methods.[1]
CTAB (0.78 g, 2.14 mmol) was dissolved in a mixture of ethanol (3.32 mL) and nanopure water (21.6 mL), followed by DEA (41.4 µL, 0.428 mmol). The solution was stirred at 60°C for 30

min. APTES (7.64 µL, 32.6 µmol) was added, followed by dropwise addition of TEOS (2.19 mL, 9.80 mmol) over 5 min. The reaction mixture was stirred for 18 h at 60°C. Nanoparticles were collected by centrifugation (13,000 rpm for 15 min), washed with ethanol (3×), and stored in ethanol.

S3.2 Surfactant Template Extraction:

MSNs were washed with a methanolic solution of 1M HCl (10 mg MSNs in 1 mL of acid solution) and stirred at 60°C for 10 h. After collecting and washing, a second acid wash was performed under identical conditions for 6 h. Surfactant-free AP-MSNs were washed with ethanol (3×) and stored in ethanol. This methodology ensures the successful synthesis of AP-MSNs suitable for prodrug loading and subsequent applications.

S3.3 Characterization of Nanomaterials

Dynamic Light Scattering (DLS) and Zeta Potential Analysis.

Hydrodynamic size measurements of mesoporous silica nanoparticles (MSNs) were carried out using a Zetasizer Nano instrument (Malvern Instruments). MSNs were dispersed at a concentration of 0.1 mg mL^{-1} in deionized water, PBS (1 mM, pH 7.4), or complete cell culture medium supplemented with 10% fetal bovine serum (FBS). The dispersions were sonicated for 10 min prior to analysis. Zeta (ζ) potential measurements were conducted using the same instrument and MSN concentration in PBS (1 mM, pH 7.4).

Surface Area and Pore Size Analysis.

MSNs (20-25 mg) were dried under vacuum and degassed overnight at 80 °C to remove physisorbed solvents. Nitrogen adsorption-desorption isotherms were obtained using a NOVA 2200e surface area and pore size analyzer (Quantachrome). Specific surface area was calculated using the Brunauer-Emmett-Teller (BET) method, while pore volume and pore diameter were determined using the Barrett-Joyner-Halenda (BJH) analysis.

S4. Synthesis of cisPt-MSNs

S4.1 cisPt-MSNs (9 %wt): AP-MSN (62 mg) was dispersed in DMSO (1.24 mL) with TEA (13.02 µL). Compound 2 (24.8 mg) and EDC (44.64 mg) were dissolved in 1.24 mL of DMSO and added to the MSN dispersion. The two solutions were combined and stirred at room temperature for 24 h. The resulting cisPt-MSNs were collected via centrifugation, washed once with DMSO, twice with ethanol, and stored in ethanol.

S4.2 cisPt-MSNs (21 %wt): AP-MSN (705 mg) was dispersed in DMSO (21.15 mL) with TEA (148.05 µL). Compound 2 (282 mg) and EDC (507.6 mg) were dissolved in 14.1 mL of DMSO and added to the MSN dispersion. The two solutions were combined and stirred at room temperature for 24 h. The resulting cisPt-MSNs were collected via centrifugation, washed once with DMSO, twice with ethanol, and stored in ethanol.

The amount of cisPt was determined by atomic absorption spectroscopy (AAS). The % of cisPt in MSNs was calculated as:

cisPt loaded (%wt) = (Mass of cisPt added – Mass of cisPt in the supernatant)/Mass of MSNs x 100

S5. Synthesis of Phosphonate-Grafted MSNs (Phos-MSNs/Phos-cisPt-MSNs)

Phosphonate-functionalized MSNs were synthesized by post-synthetic grafting with trimethylphosphite (TPMP). AP-MSNs or cisPt-MSNs (200 mg) were dispersed in nanopure water (13 mL), and an aqueous solution of TPMP (113.5 µL, 0.2 mmol) was added (pH adjusted to 6-7). The mixture was stirred at 40°C for 3 h. The nanoparticles were then collected via centrifugation and washed thrice with ethanol to obtain Phos-MSNs or Phos-cisPt-MSNs.

S6. Synthesis of PEI coated MSNs

Polyethyleneimine (PEI, MW = 1.8 kDa) coating was performed on Phos-MSNs or Phos-cisPt-MSNs to enhance stability and facilitate further functionalization. Phos-MSNs or Phos-cisPt-MSNs (100 mg) were dispersed in ethanol (40 mL), and a solution of PEI (10 mL, 2.5 mg mL^{-1} in ethanol) was added. The suspension was stirred for 1 h at room temperature. The nanoparticles were collected via centrifugation and washed thrice with ethanol to yield PEI-MSNs or PEI-cisPt-MSNs.
To quantify the PEI coating, a ninhydrin assay was performed. PEI-MSNs (1 mg) were dispersed in 4 mL of ethanol, and 1 mL of ninhydrin reagent (15 mg mL^{-1} in ethanol) was added. The reaction was stirred for 24 h, and the supernatant absorbance was measured at 575 nm using UV-Vis spectroscopy. A calibration curve was generated using known concentrations of PEI.

S7. Synthesis of Gem-MSNs (10 and 18 %wt)

To synthesize Gem-MSNs (18 %wt), SPDP-MSNs (30 mg) were dispersed in 10 mL of methanol. A solution of Gem prodrug (30 mg, 85.4 µmol) in 5 mL of methanol was added to the dispersion, and the reaction mixture was stirred at room temperature for 72 h. After the final conjugation step, the nanoparticles were collected via centrifugation, washed once with methanol, followed by a wash with ethanol, and stored for further use.

To synthesize Gem-MSNs (10 %wt), the reaction was done adding 20–25 mg of Gem prodrug to the reaction mixture and stirred for 48 hours. After the final conjugation step, the nanoparticles were collected via centrifugation, washed once with methanol, followed by a wash with ethanol, and stored for further use.

S8. Synthesis of Gem-cisPt-MSNs

S8.1 Gem-cisPt-MSN (10:9 %wt): SPDP-MSNs or SPDP-cisPt-MSNs (9 %wt) (30 mg) were dispersed in methanol (10 mL). A solution of Gem prodrug (30 mg, 85.4 µmol) in 5 mL methanol was added. The mixture was stirred for 72 h, and the resulting nanoparticles were collected via centrifugation, washed with methanol and ethanol, and stored.

S8.2 Gem-cisPt-MSN (18:9 %wt) and Gem-cisPt-MSN (18:21 %wt): Following the initial reaction for 10% loading of Gem, an additional 20-25 mg of Gem prodrug was added and stirred for 72 h or stirring time was increased to 6 days to achieve 18% loading.

S9. Quantification of Gem loading

The conjugation efficiency of Gem was determined by measuring the amount of 2-thiopyridine, a byproduct of the disulfide exchange reaction between Gem prodrug and SPDP-MSNs. The supernatants from the reaction and washing steps were analyzed for 2-thiopyridine content using UV-Vis spectroscopy at 357 nm. The %wt loaded of Gem was determined according to the equation below. This systematic synthesis provides tunable ratios of gemcitabine and cisplatin loading on MSNs, optimizing their potential for combined cancer therapy.

Gem loaded (%wt) = (Mass of Gem added – Mass of Gem in the supernatant)/Mass of MSNs x 100

S10. Internalization experiments

AsPC-1 and GR-BxPC3 cells were plated in 6-well plates at seeding densities of $1.5x10^5$ and 2.5 $x10^5$ cells per well, respectively, to accommodate their distinct growth rates. After plating, cells were incubated for 24 h to allow them to reach optimal confluency (typically ~70–80%) and ensure nutrient-replete growth in the logarithmic phase. Subsequently, cells were treated with five nanoparticle formulations with the concentration of 5 µg/mL per well: Gem-MSN (10 %wt); Gem-MSN (18 %wt); Gem-cisPt-MSN (10:9 %wt); Gem-cisPt-MSN (18:9 %wt); Gem-cisPt-MSN (18:21 %wt). After a 10h incubation, internalization was assessed using flow cytometry. All the conditions were performed in triplicate (n=3) and reported as mean ± SD.

AsPC1 and GR-BxPC3 cells were seeded on 6-well plates with coverslips with a cell density of 1.5 x 10^5 and 2.5 $x10^5$ cells per well respectively and incubated for 24h to allow cells to adhere to coverslips. After 24h, wells were washed with PBS, and treated with five FITC tagged nanoparticle formulations Gem-MSN (10 %wt); Gem-MSN (18 %wt); Gem-cisPt-MSN (10:9 %wt); Gem-cisPt-MSN (18:9 %wt); Gem-cisPt-MSN (18:21 %wt) at a concentration of 5 µg/mL per well and incubated for 10 h. Wells were then washed with PBS and nuclei were stained with NucBlue Live Cell Stain Ready Probes Reagent for 15 min. Grace Bio-Labs SecureSeal™ imaging spacers were added to slides, followed by media and coverslips.
Images were taken using the Leica Stellaris 8 confocal microscope at a resolution of 1024 x 1024 pixels. Live cells were imaged with nuclei staining of NucBlue at 405 nm excitation wavelength and FITC 495 nm excitation wavelength at 40x magnification. Nuclei staining at 405 nm was imaged at 2.5% smart gain and 7.6% intensity for BxPC3 cells and 16.1% smart gain and 8.0% intensity for AsPC1 cells. FITC staining at 495 nm was imaged at 10% smart gain and 19% intensity.

S11. Western Blot Image Analysis

For analysis, the β-actin bands for each sample were used to normalize with the proteins of interest using ImageLab by Bio-Rad. The adjusted volume (intensity) of β-actin and protein of

interest was measured and a ‘local’ background subtracted was considered by the software in the adjusted volume. The values for protein of interest were divided by β-actin and the trends of C/GR samples were determined.

S12. Quantitative Real-Time PCR (qRT-PCR)

Quantitative RT-PCR was performed on a QuantStudio 3 (Applied Biosystems). Primers used for GAPDH, ENT1, dCK, and RRM1 amplification are listed below. The GAPDH, ENT1, and dCK primers adapted from Vena et al., 2020[2] and the RRM1 primer set was designed using PrimerQuest Tool (Integrated DNA Technologies) for BxPC3 cells. For KCM cells, GAPDH (PrimerBank ID 126012538c2), ENT1 (Slc29a1, PrimerBank ID 12584968a1), and dCK(PrimerBank ID 124286803c2), were purchased and RRM1 primer set was designed using PrimerQuest Tool (Integrated DNA Technologies).

The primers used for qPCR are listed for each gene targeted in BxPC3 and KCM cells.

**Gene, Forward (F) or Reverse (R)**	**Target sequence for KCM 5’ -> 3’**	**Target sequence for BxPC3 5’ -> 3’**
GAPDH-F	TGACCTCAACTACATGGTCTACA	TCACCAGGGCTGCTTTTAAC
GAPDH-R	*CTTCCCATTCTCGGCCTTG*	ATCTCGCTCCTGGAAGATGG
dCK-F	GAAGAGCGGTGGAAATGTTCT	GCTGCAGGGAAGTCAACATT
dCK-R	GCATCTTTGAGCTTGCCATTG	TCAGGAACCACTTCCCAATC
ENT1-F	*CAGCCTCAGGACAGGTATAAGG*	TCTTCTTCATGGCTGCCTTT
ENT1-R	*GTTTGTGAAATACTTGGTTGCGG*	CCTCAGCTGGCTTCACTTTC
RRM1-F	CTACGGTTGGAAGCAGGGTT	GCAGCTGAGAGAGGTGCTTT
RRM1-R	GCTGCTGTGTTCCTCTCCTT	CAGGATCCACACATCAGACA

S13. Confocal Microscopy

Images were taken on the confocal microscope using the 40x magnification a 1x zoom and formatted at a resolution of 1024x1024 pixels. The nuclei staining was imaged at 405 nm excitation wavelength (DAPI channel), while the target proteins of Vimentin, N-cadherin, and E-cadherin were imaged at 578 nm excitation wavelength. For BxPC3 cells, Vimentin and DAPI stained images at 578 and 405 nm respectively were imaged at 1.6% intensity with 2.5% smart gain. N-cadherin stained BxPC3 cells were imaged at 14.08% intensity with a smart gain of 9.3% at 578 nm with DAPI at 405 nm imaged at 1.9% intensity and 2.5% smart gain. E-cadherin stained BxPC3 cells were measured at 6.1% intensity with a smart gain of 7% with DAPI set to 1.41% at 2.5% smart gain. For KCM cells, DAPI at 405 nm was measured at 0.89% intensity

with 2.5% smart gain and Vimentin at 578 nm was measured at 4.77% intensity with 2.5% smart gain. N-cadherin for KCM cells were measured at 16.8% with a smart gain of 12% with DAPI set to 1.16% at 2.7% smart gain. E-cadherin KCM cells were measured at 6.32% with a smart gain of 12.4% with DAPI set to 1.58% at 2.5% smart gain.

S14. Flow cytometry experiments for ROS generation

C-KCM/GR-KCM cells were seeded at 7.0×10^4 cells/well in 6-well plates and incubated for 24h at 37 ^oC with 5% CO_2 in a humidified chamber. For all BxPC3 subtypes, the seeding density was 6.0×10^5 cells/well. All cells were treated for 12h with Gem- and/or cisPt-loaded MSNs, as well as PEI-MSNs, using the same concentrations applied in the apoptosis and cell-cycle studies. After treatment for the indicated time point, cells were washed once with cold PBS and incubated with serum-free medium containing 10 µM 2',7'-dichlorodihydrofluorescein diacetate (DCFDA) for 30 min at 37 °C in the dark. Cells were then washed three times with cold PBS, trypsinized, harvested and centrifuged to remove residual trypsin. The resulting cell pellets were washed again with cold PBS and resuspended in PBS for flow cytometric analysis. Fluorescence was acquired using the FITC channel, and ROS levels were quantified as the percentage of DCF-positive cells relative to the unstained population.

Supplementary Tables

Table S1. IC_{50} values of free Gem for KCM and BxPC3 cells.

**PDAC cells**	**IC_{50} (nM)**
C-KCM	6.9 ± 1.9
GR-KCM	61.5 ± 6.4
C-BxPC3	56.5 ± 7.7
GR-BxPC3	166.9 ± 26.0

Table S2. Drug content in various ratios of Gem-cisPt-MSNs (% wt).

**Drug Combinations**	**Loading (%) of cisPt** (n=4)	**Loading (%) of Gem** (n=4)	**Molar ratio (cisPt:Gem)**
Gem-MSNs	_	18.7 ± 0.7	_
Gem-MSNs	_	10.9 ± 1.3	_

cisPt-MSNs	21.1 ± 1.2	_	_
Gem- cisPt -MSNs	21.1 ± 1.2	18.7 ± 0.7	1.20:1
cisPt-MSNs	9.2 ± 3.4	_	_
Gem- cisPt -MSNs	9.2 ± 3.4	10.9 ± 1.3	0.93:1
Gem- cisPt -MSNs	9.2 ± 3.4	18.7 ± 0.7	0.53:1

Table S3. Pore-surface Analysis Data of nanomaterials (BET-BJH method).

Material	Surface Area (m2/g) (BET)	Pore Volume (cc/g) (BJH)	Pore-Diameter (nm) (BJH)
Unmodified AP-MSNs (n=12)	799±77.0 (n=8)	1.45±0.2 (n=8)	2.3±0.1 (n=8)
After functionalization AP-MSNs (n=12)	782±52 (n=4)	1.0±0.2 (n=4)	2.1±0.1 (n=4)

Table S4: Dynamic Light Scattering Analysis Data of nanomaterials.

Dynamic Light Scattering Analysis Data			
**Type of Materials**	**Polydispersity Index (n=08)**	**Z Avg (d, nm) (n=08) in PBS**	**Zeta Potential (mV) (n=08) in PBS**
AP-MSNs	0.16±0.03	107.6±15	-33.2±2.3
cisPt-MSN	0.16±0.03	140.3±4	-37.1±4.1
Phos-cisPt-MSN	0.09±0.01	91.4±5	-45.6±3.6
PEI-cisPt-MSN	0.3±0.04	554.23±4	38.1±2.1

SPDP-cisPt-MSN	0.74±0.1	667.3±11	32.11±3.2
Gem-MSNs (10 %wt)	0.21±0.04	167±4.3	21±2.31
Gem-MSN (18 %wt)	0.28±0.01	220±7	17.30±4.1
Gem-cisPt-MSN (10:9 %wt)	0.32±0.02	310±3	33.22±3.3
Gem-cisPt-MSN (18:9 %wt)	0.34±0.015	323±4	28.40±4.1
Gem-cisPt-MSN (18:21 %wt)	0.24±0.014	315±5	31.43±1.1

Note: For all the IC_{50} values, we will only report the concentrations in **μg/mL and average of all replicates in nM.**

Table S5. IC_{50} values in various ratios of Gem-cisPt-MSNs (KCM and BxPC3 Cell lines).

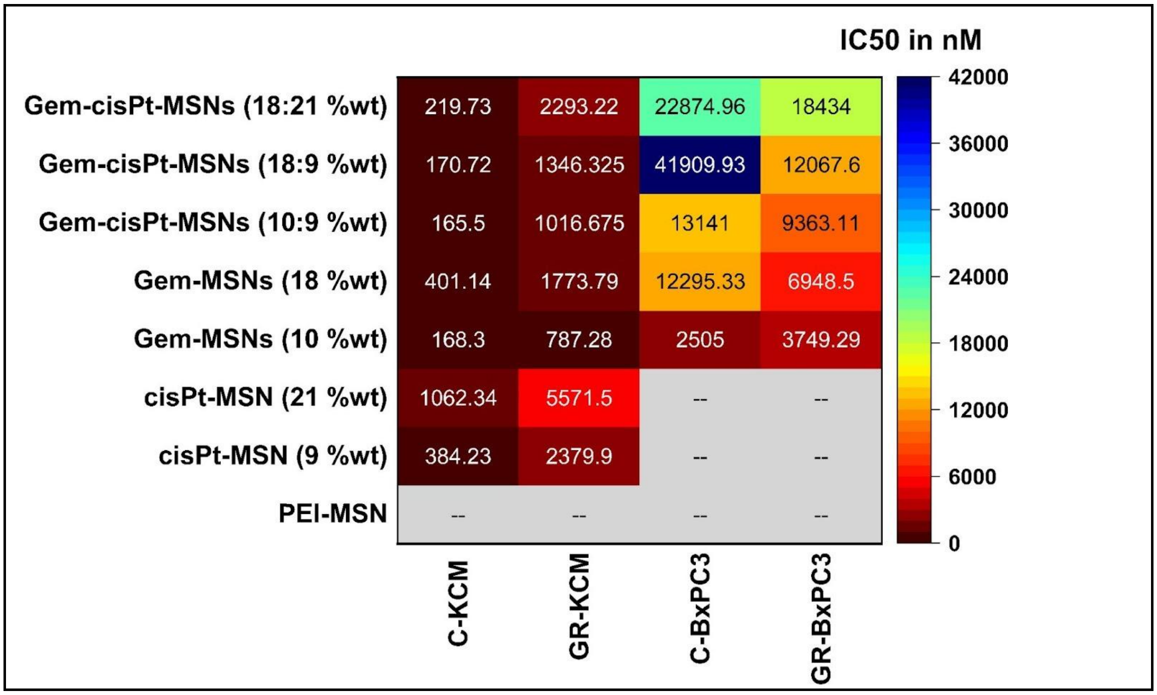

IC50 in nM

	C-KCM	GR-KCM	C-BxPC3	GR-BxPC3
Gem-cisPt-MSNs (18:21 %wt)	219.73	2293.22	22874.96	18434
Gem-cisPt-MSNs (18:9 %wt)	170.72	1346.325	41909.93	12067.6
Gem-cisPt-MSNs (10:9 %wt)	165.5	1016.675	13141	9363.11
Gem-MSNs (18 %wt)	401.14	1773.79	12295.33	6948.5
Gem-MSNs (10 %wt)	168.3	787.28	2505	3749.29
cisPt-MSN (21 %wt)	1062.34	5571.5	--	--
cisPt-MSN (9 %wt)	384.23	2379.9	--	--
PEI-MSN	--	--	--	--

Table S6. IC_{50} values in various ratios of Gem-cisPt-MSNs (AsPC1 and HPAFII Cell lines).

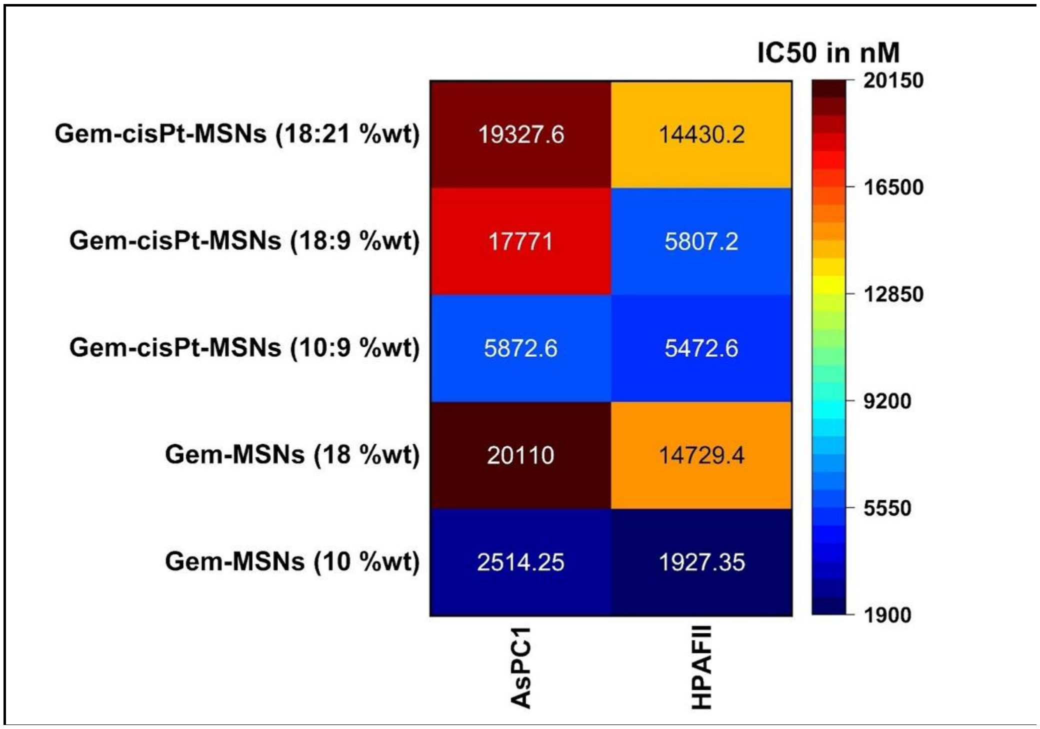

Table S7. Cell cycle analysis for C-KCM and GR-KCM cell subtype after cisPt and/or Gem-MSNs treatment.

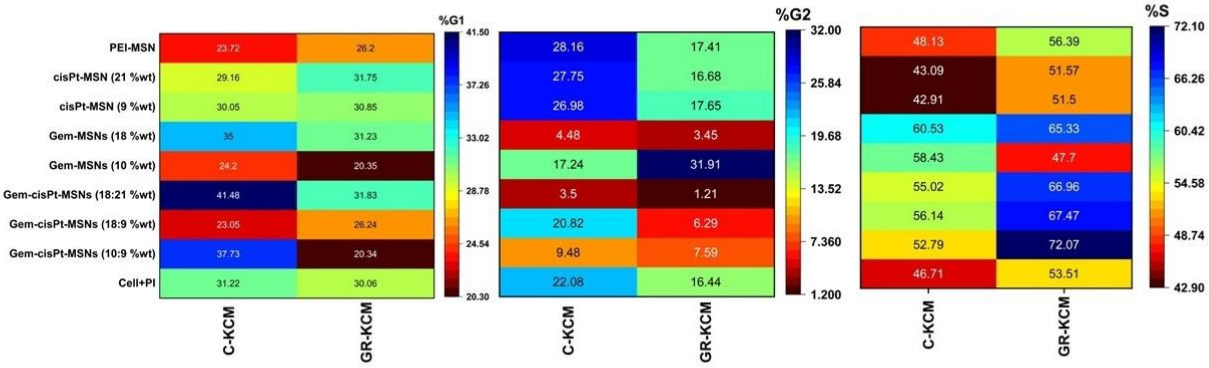

Table S8. Cell cycle analysis for C-BxPC3 and GR-BxPC3 cell subtype after cisPt and/or Gem-MSNs treatment.

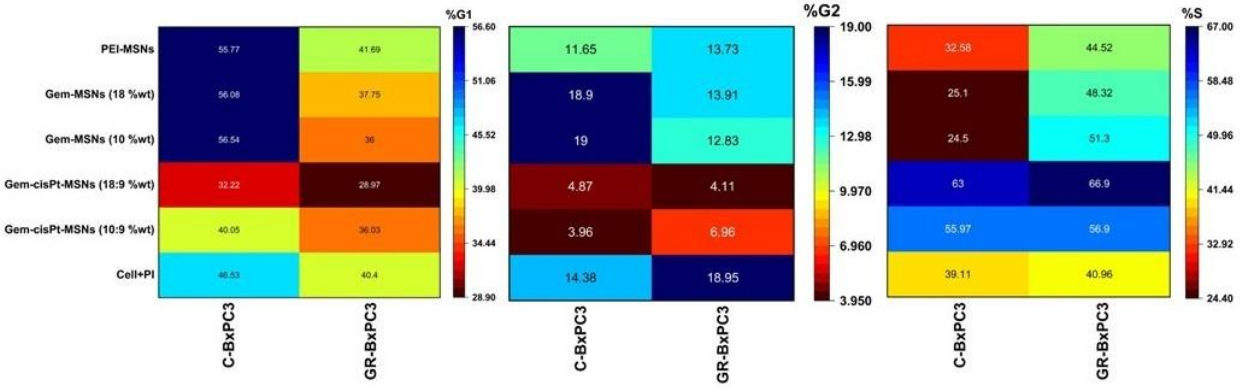

Table S9. Percentage of apoptotic KCM and BxPC3 cells after cisPt and/or Gem-MSNs treatment.

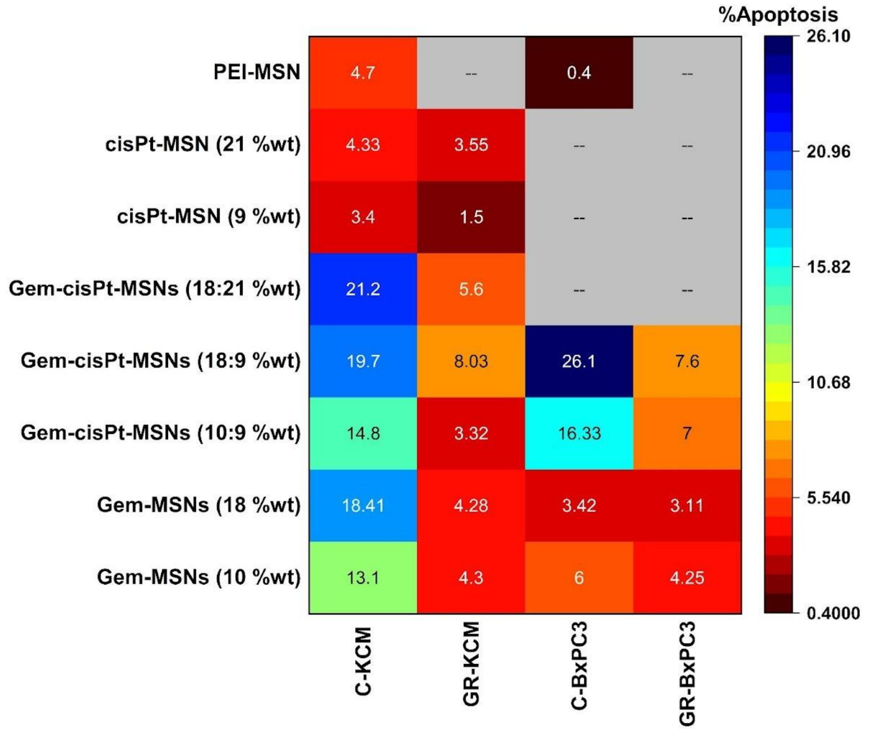

TableS10: Time-dependent intracellular ROS generation induced by Gem- and/or cisPt-loaded MSNs. (A) GR-KCM cells, (B) C-KCM cells, (C) GR-BxPC3 cells, and (D) C-BxPC3 cells

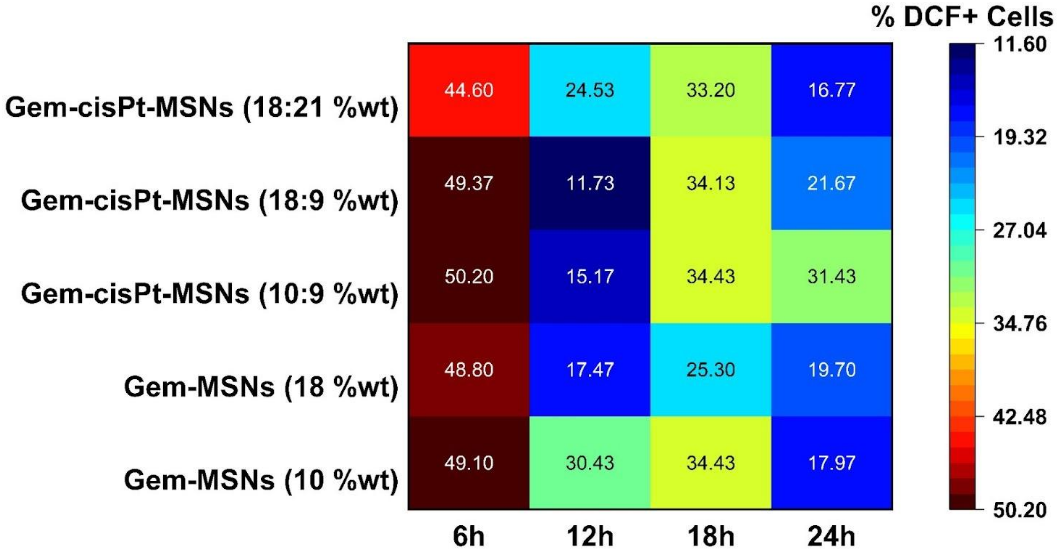

% DCF+ Cells
Gem-cisPt-MSNs (18:21 %wt)
Gem-cisPt-MSNs (18:9 %wt)
Gem-cisPt-MSNs (10:9 %wt)
Gem-MSNs (18 %wt)
Gem-MSNs (10 %wt)
6h
12h
18h
24h
44.60 24.53 33.20 16.77
49.37 11.73 34.13 21.67
50.20 15.17 34.43 31.43
48.80 17.47 25.30 19.70
49.10 30.43 34.43 17.97
11.60
19.32
27.04
34.76
42.48
50.20

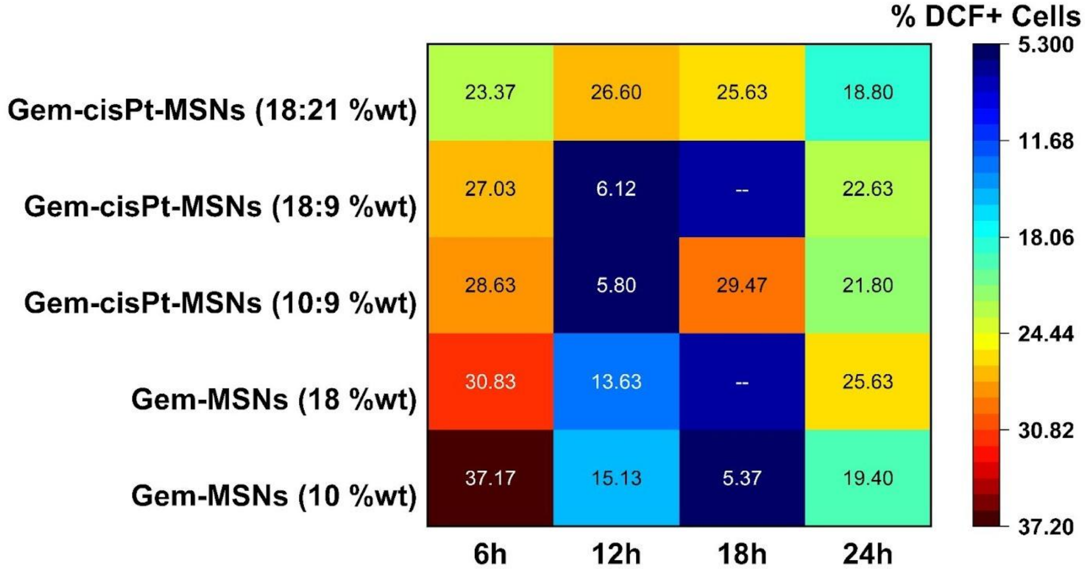

% DCF+ Cells
Gem-cisPt-MSNs (18:21 %wt)
Gem-cisPt-MSNs (18:9 %wt)
Gem-cisPt-MSNs (10:9 %wt)
Gem-MSNs (18 %wt)
Gem-MSNs (10 %wt)
6h
12h
18h
24h
23.37 26.60 25.63 18.80
27.03 6.12 -- 22.63
28.63 5.80 29.47 21.80
30.83 13.63 -- 25.63
37.17 15.13 5.37 19.40
5.300
11.68
18.06
24.44
30.82
37.20

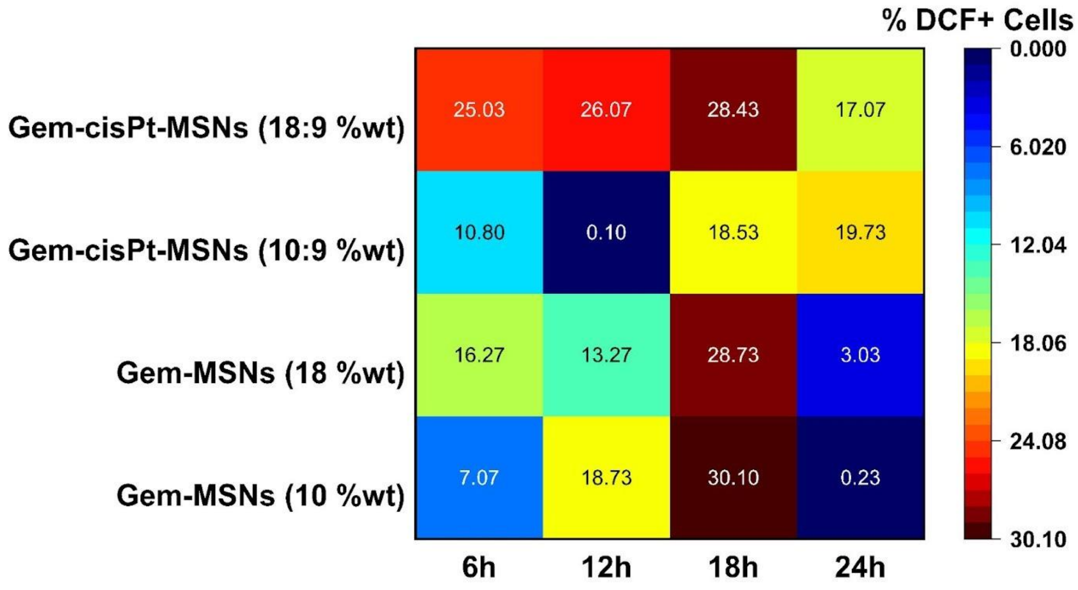

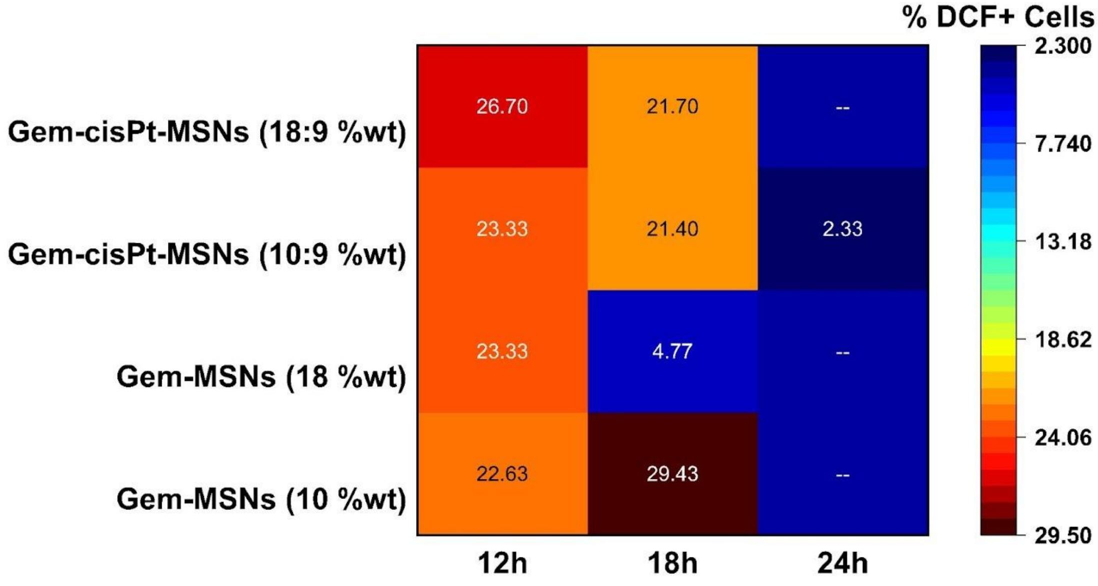

Supplementary Figures

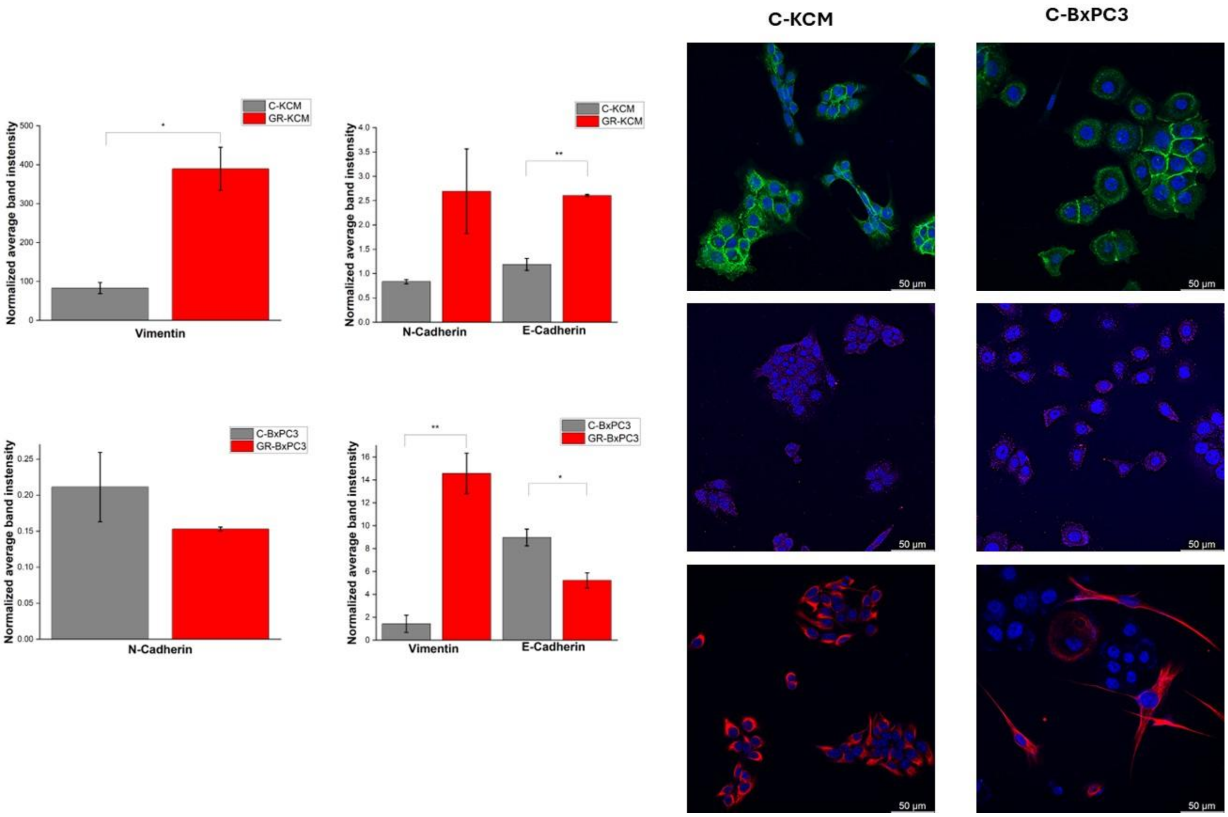

Figure S1. Expression of EMT markers in KCM and BxPC3 cells. Quantification of protein levels of Vimentin, N- and E0cadherin in C/GR-KCM and C/GR-BxPC3 cells (left) and EMT marker expression in C-KCM and C-BxPC3 cells. Statistical comparisons were conducted using one-way ANOVA followed by Tukey's multiple comparison test. Significance levels are denoted as follows: $**p \leq 0.01$, and $*p \leq 0.05$.

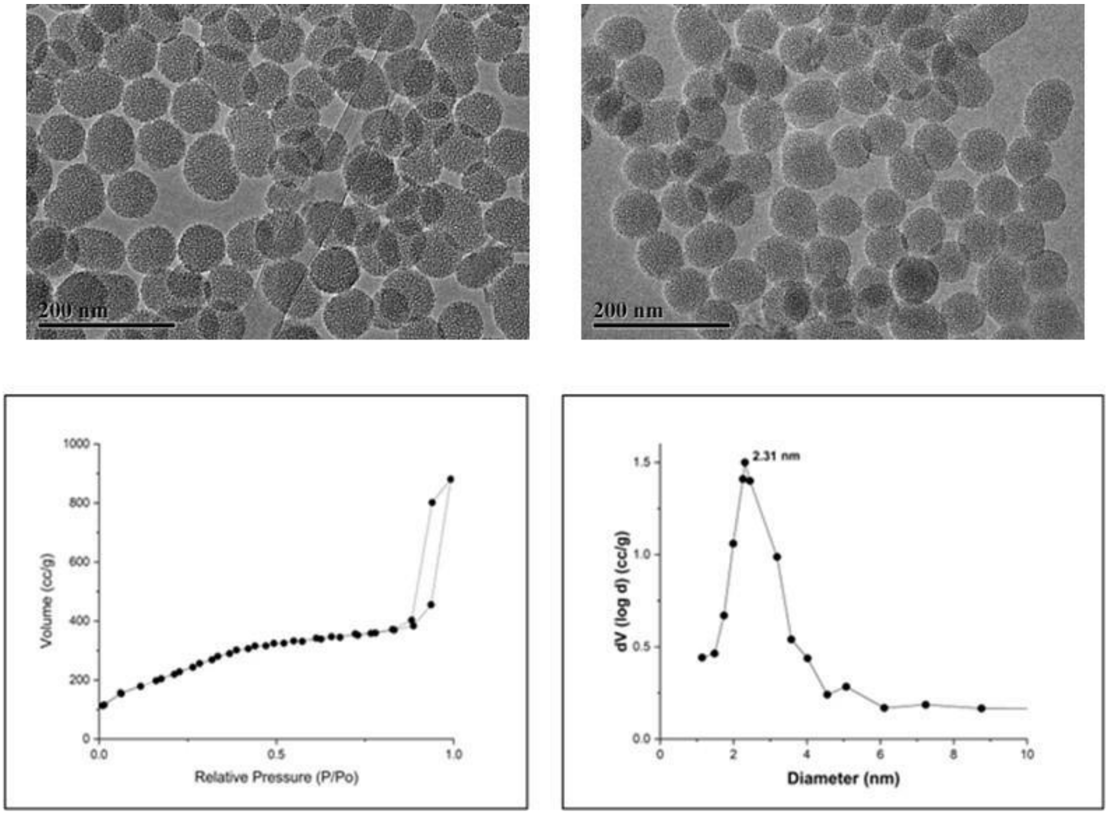

Figure S2. Nitrogen adsorption-desorption isotherms and pore size distribution of mesoporous silica nanoparticles (MSNs).

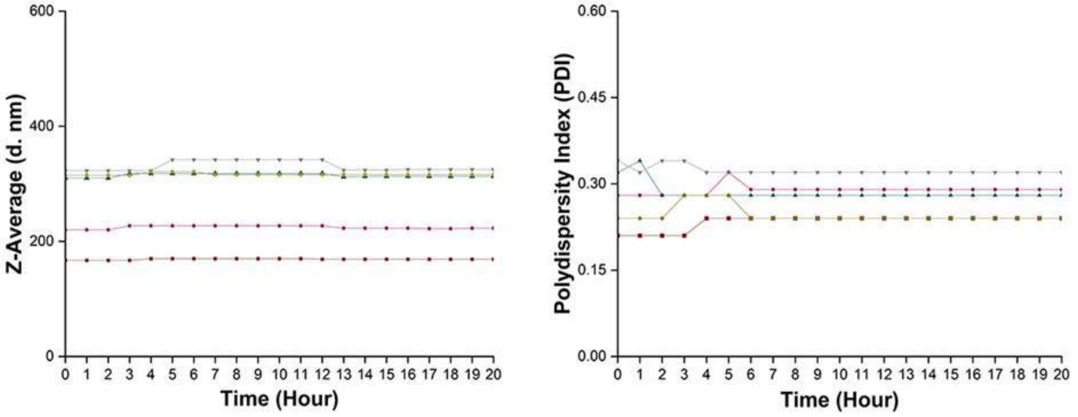

Figure S3. Evaluation of long-term colloidal stability (Cell media) of the formulations through Z-average hydrodynamic diameter and PDI-tracking over time, demonstrating the stability of nanoparticles under storage or physiological conditions. Color code: AP-MSNs (Cyan); cisPt-MSN (Lime Green); Phos-cisPt-MSN (Burnt Orange); PEI-cisPt-MSN (Navy Blue); SPDP-cisPt-MSN (Lavender); Gem-MSN (10 %wt) (Brick Red); Gem-MSN (18 %wt) (Burgundy);

Gem-cisPt-MSN (10:9 %wt) (Dark Green); Gem-cisPt-MSN (18:9 %wt) (Gray); Gem-cisPt-MSN (18:21 %wt) (Olive).

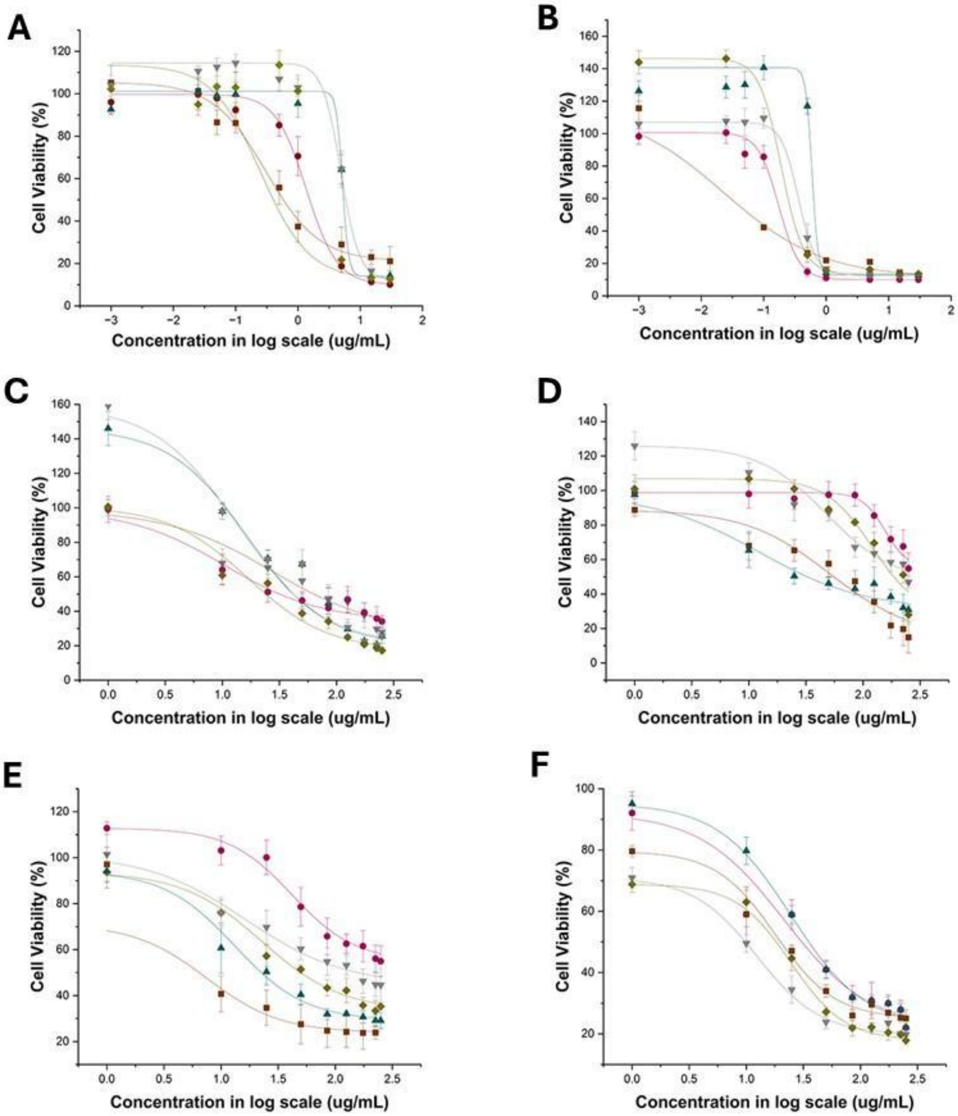

Figure S4. Dose-response curves of mono- and dual-drug loaded MSNs across pancreatic cancer cell lines. **A)** GR-KCM, **B)** C-KCM, **C)** GR-BxPC3, **D)** C-BxPC3, **E)** AsPC1 and **F)** HPAF-II. Error bars represent the standard deviation of three biological replicates (n=3). Color code: AP-MSNs (Cyan); cisPt-MSN (Lime Green); Phos-cisPt-MSN (Burnt Orange); PEI-cisPt-MSN (Navy Blue); SPDP-cisPt-MSN (Lavender); Gem-MSN (10 %wt) (Brick Red); Gem-MSN (18 %wt) (Burgundy); Gem-cisPt-MSN (10:9 %wt) (Dark Green); Gem-cisPt-MSN (18:9 %wt) (Gray); Gem-cisPt-MSN (18:21 %wt) (Olive).

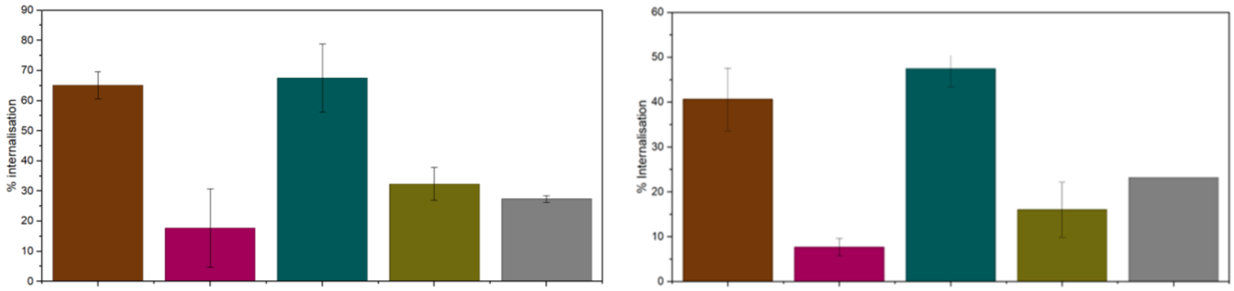

Figure S5. Internalization of Gem monotherapy (Gem-MSNs = 10 and 18 %wt) and the combinations (Gem-cisPt-MSNs = 10:9, 18:9 and 18:21 %wt) were evaluated in GR-BxPC3 (left) and AsPC1 (right) cells using flow cytometry. Gem-MSN (10 %wt) (Brick Red); Gem-MSN (18 %wt) (Burgundy); Gem-cisPt-MSN (10:9 %wt) (Dark Green); Gem-cisPt-MSN (18:9 %wt) (Gray); Gem-cisPt-MSN (18:21 %wt) (Olive). All data are presented as mean ± standard deviation (SD) from three independent biological replicates (n=3).

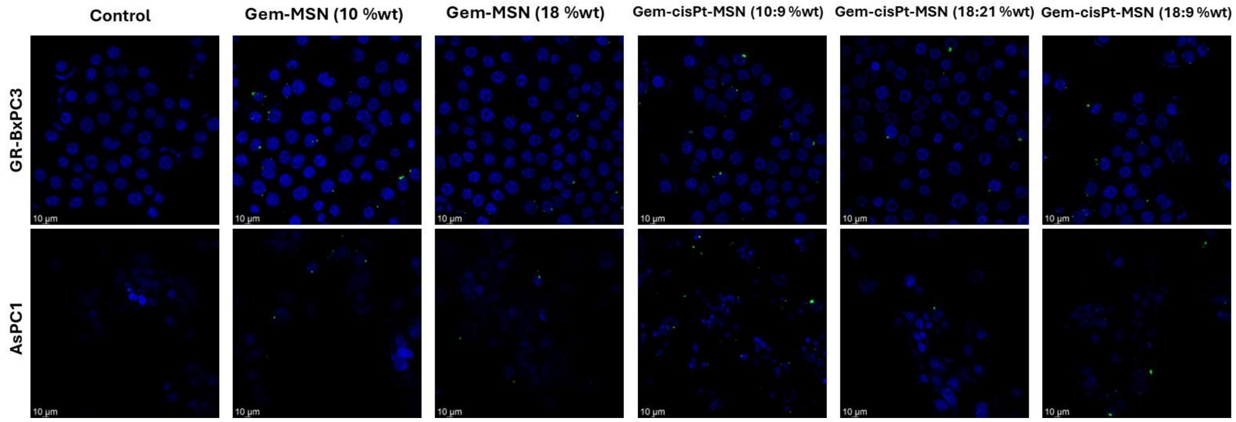

Figure S6. Live-cell imaging with internalization of MSNs in GR-BxPC3 and AsPC1 cells at 40x magnification. Nuclei-stained blue using NucBlue and MSNs-FITC conjugated stained as green.

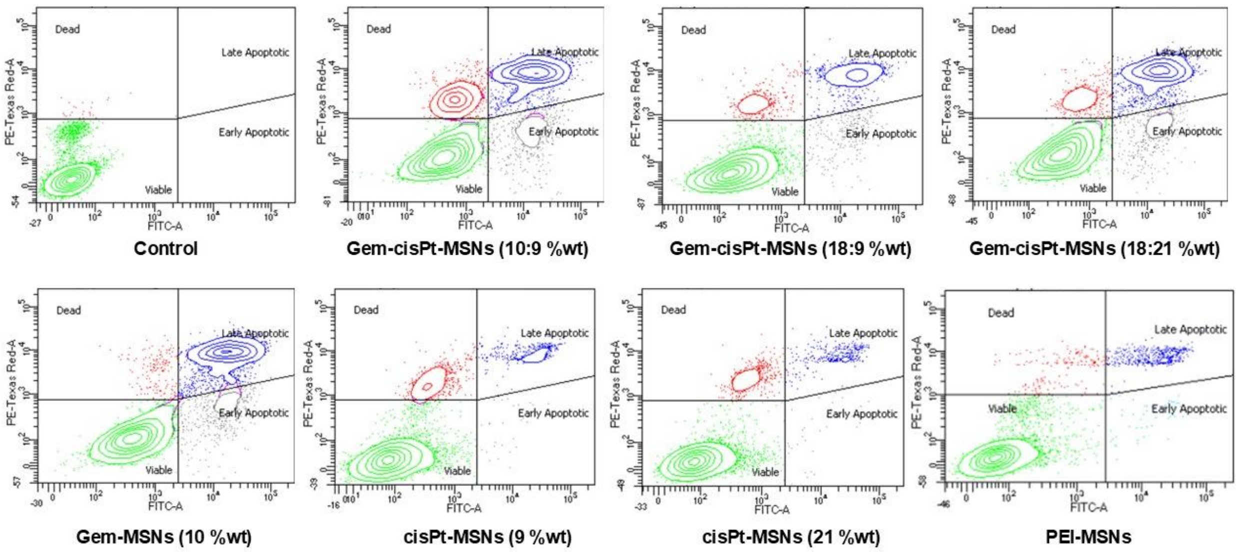

Figure S7. Flow cytometry analysis was performed following treatment with various mesoporous silica nanoparticle (MSN) formulations. Based on Annexin V-FITC and propidium iodide (PI) staining, cell populations were categorized as follows: Annexin V-FITC^{-}/PI^{-} (FITC^{-}/PI^{-}) cells were considered viable; Annexin V-FITC^{+}/PI^{-} (FITC^{+}/PI^{-}) cells were classified as early apoptotic; Annexin V-FITC^{+}/PI^{+} (FITC^{+}/PI^{+}) cells were indicative of late apoptosis; and Annexin V-FITC^{-}/PI^{+} (FITC^{-}/PI^{+}) cells were identified as dead. Data represents the mean ± SD of three independent experiments (n=3).

Supplementary references

(1) Tarannum, M.; Hossain, M. A.; Holmes, B.; Yan, S.; Mukherjee, P.; Vivero-Escoto, J. L. Advanced Nanoengineering Approach for Target-Specific, Spatiotemporal, and Ratiometric Delivery of Gemcitabine-Cisplatin Combination for Improved Therapeutic Outcome in Pancreatic Cancer. *Small* **2022**, *18* (2). DOI: 10.1002/smll.202104449.

(2) Vena, F.; Causi, E. L.; Rodriguez-Justo, M.; Goodstal, S.; Hagemann, T.; Hartley, J. A.; Hochhauser, D. The MEK1/2 Inhibitor Pimasertib Enhances Gemcitabine Efficacy in Pancreatic Cancer Models by Altering Ribonucleotide Reductase Subunit-1 (RRM1). *Clinical Cancer Research* **2015**, *21* (14), 5563–5577. DOI: 10.1158/1078-0432.CCR-15-0485.
